# Identification and Mode of Action of a Plant Natural Product Targeting Human Fungal Pathogens

**DOI:** 10.1128/AAC.00829-17

**Published:** 2017-08-24

**Authors:** Stéphane Dorsaz, Tiia Snäkä, Quentin Favre-Godal, Pierre Maudens, Nathalie Boulens, Pascal Furrer, Samad Nejad Ebrahimi, Matthias Hamburger, Eric Allémann, Katia Gindro, Emerson Ferreira Queiroz, Howard Riezman, Jean-Luc Wolfender, Dominique Sanglard

**Affiliations:** a Institute of Microbiology, University of Lausanne, and Lausanne University Hospital, Lausanne, Switzerland; b School of Pharmaceutical Sciences, EPGL, University of Geneva-University of Lausanne, Geneva, Switzerland; c Department of Biochemistry, NCCR Chemical Biology, University of Geneva, Geneva, Switzerland; d Agroscope, Strategic Research Division Plant Protection, Mycology, and Biotechnology, Nyon, Switzerland; e Division of Pharmaceutical Biology, Department of Pharmaceutical Sciences, University of Basel, Basel, Switzerland

**Keywords:** antifungal agents, natural products, sterol biosynthesis, antifungal therapy

## Abstract

Candida albicans is a major cause of fungal diseases in humans, and its resistance to available drugs is of concern. In an attempt to identify novel antifungal agents, we initiated a small-scale screening of a library of 199 natural plant compounds (i.e., natural products [NPs]). *In vitro* susceptibility profiling experiments identified 33 NPs with activity against C. albicans (MIC_50_s ≤ 32 μg/ml). Among the selected NPs, the sterol alkaloid tomatidine was further investigated. Tomatidine originates from the tomato (Solanum lycopersicum) and exhibited high levels of fungistatic activity against Candida species (MIC_50_s ≤ 1 μg/ml) but no cytotoxicity against mammalian cells. Genome-wide transcriptional analysis of tomatidine-treated C. albicans cells revealed a major alteration (upregulation) in the expression of ergosterol genes, suggesting that the ergosterol pathway is targeted by this NP. Consistent with this transcriptional response, analysis of the sterol content of tomatidine-treated cells showed not only inhibition of Erg6 (C-24 sterol methyltransferase) activity but also of Erg4 (C-24 sterol reductase) activity. A forward genetic approach in Saccharomyces cerevisiae coupled with whole-genome sequencing identified 2 nonsynonymous mutations in *ERG6* (amino acids D249G and G132D) responsible for tomatidine resistance. Our results therefore unambiguously identified Erg6, a C-24 sterol methyltransferase absent in mammals, to be the main direct target of tomatidine. We tested the *in vivo* efficacy of tomatidine in a mouse model of C. albicans systemic infection. Treatment with a nanocrystal pharmacological formulation successfully decreased the fungal burden in infected kidneys compared to the fungal burden achieved by the use of placebo and thus confirmed the potential of tomatidine as a therapeutic agent.

## INTRODUCTION

Invasive fungal infections are an increasing threat to human health. In developed countries, these infections predominantly occur in the context of increasingly aggressive immunosuppressive therapies. The overall mortality from invasive diseases caused by Candida and Aspergillus spp. is 30 to 50%, despite the advent of new diagnostic and therapeutic strategies (1). The fight against Candida albicans infections necessitates the use of antifungal agents, and continued efforts are required to improve the therapeutic outcomes associated with fungal infections.

Antifungal drugs that are currently available for the treatment of Candida infections belong to four different chemical classes and include polyenes, azoles, pyrimidine analogues, and echinocandins (2). While polyenes and azoles target sterols and their biosynthesis, pyrimidine analogues perturb nucleic acid biosynthesis and echinocandins interfere with cell wall biosynthesis. The activity against common fungal pathogens and their detailed mode of action are summarized in available reviews (3, 4). The repeated or long-term use of antifungal agents in medicine has facilitated the development of resistance in clinically relevant species (5). When it occurs, antifungal resistance can be a serious clinical problem due to the limited number of available agents. In general, the incidence of antifungal resistance among human fungal pathogens is low to moderate, especially compared to the incidence of antibiotic resistance among bacterial pathogens. Antifungal resistance occurrence must be considered independently for each antifungal class and for each fungal species. Moreover, epidemiological data regarding the incidence of resistance among fungal species are not identically distributed worldwide (6, 7). Taken together, the small number of available antifungal agents and the occurrence of resistance reveal the urgent need for novel active compounds.

Natural products (NPs) have already provided a vast resource for active ingredients in medicines. The reason for this success can be explained by the high chemical diversity of NPs, the effects of evolutionary pressure to create biologically active molecules, and the structural similarity of protein targets across many species (8). In the field of antimicrobials, NPs have met with important successes. Starting with the discovery of penicillin, the pharmaceutical industry has relied on this source extensively for antibiotic development. Nowadays, 80% of all available clinically used antibiotics are directly (or indirectly) derived from NPs (9). Some antifungals, including polyenes and echinocandins, derive directly from NPs.

The discovery of structurally novel NPs with suitable pharmacological properties as antibiotic leads has progressed weakly in recent decades (10). Innovative strategies have provided comprehensive profiles of the antifungal characteristics of given NPs and an understanding of their mode of action for target identification and validation (11).

In a precedent study, we reported on a strategy to identify antifungal NPs from plant crude extracts (12). This strategy relied on the use of a C. albicans isolate highly susceptible to growth inhibitors and in which traces of inhibitory NPs could be detected. NPs were identified by a bioassay that could be used as a tool enabling the rapid detection of antifungal activity. With the determination of the chemical structures of the identified NPs, novel compounds could be readily processed for further evaluation by *in vivo* approaches (13).

In this study, we report on a small-scale screening of selected NPs and an in-depth characterization of their biological properties. The compounds were tested on the basis of their activity against different pathogenic and nonpathogenic yeasts and of their toxicity for mammalian cells. One of the promising compounds (tomatidine) showing a high level of activity against C. albicans was further investigated. The tomatidine mode of action was characterized in-depth for the first time, and its activity was confirmed *in vivo*.

## RESULTS

### Screening of a small-scale library of plant NPs for antifungal activities.

A library of 199 natural products (NPs) with potential antifungal activity was built. Compounds were selected either according to their previously reported activities or by their structural analogy to scaffolds that were known to be active. These compounds were obtained in two ways: either by targeted isolation from plant extracts (29 different plants provided 53% of the investigated NPs were investigated) or by commercial acquisition after selection on the basis of their structural similarity to documented antifungals (see Materials and Methods; the compounds are listed in Table S1 in the supplemental material). The 199 NPs were subjected to standard *in vitro* microdilution susceptibility assays (EUCAST method) with C. albicans under acidic and neutral conditions (pH 4.6 and 7.0, respectively). These different values were chosen to reflect the pHs in the different host niches of C. albicans. The results are summarized in Table 1. When a threshold for antifungal activity of 32 μg/ml was considered, our analysis identified 33 NPs exhibiting antifungal activities. The activity threshold of 32 μg/ml was selected since we estimated that setting a high threshold for a MIC value *in vitro* would be problematic when activities *in vivo* were tested and an attempt to achieve therapeutic concentration ranges in animals was made. With this threshold, while 2 compounds were active at neutral pH and 18 were active only at a low pH, 13 were active under both pH conditions (Table 1).

**TABLE 1:** The 33 NPs with their biological activity profiles

Name	No.	Source	CAS^*a*^ registry no.	MIC^*b*^ (μg/ml)				LD_50_ by cytotoxicity assay	Selectivity index^*e*^
				C. albicans		C. albicans biofilm	E. coli		
				pH 7	pH 4.6				
Morindone	1	Morinda tomentosa	478-29-5	32	>32	6.25	>64	ND^*d*^	ND
Lucidine ω-methyl ether	2	Morinda tomentosa	NA^*c*^	>32	32	NA	>64	ND	ND
Morindoquinone	3	Morinda tomentosa	New NP	>32	16	25	>64	>100	6.25
Avocadene	4	Persea americana	24607-08-7	16	8	25	>64	50	6.25
Plumbagin	5	Sigma-Aldrich	481-42-5	2	4	50	>64	100	50
Alpha-hederin	6	Schefflera systila	27013-91-8	16	4	50	>64	100	6.25
Glc-3 medicagenic acid	7	Dolichos kilimandscharicus	49792-23-6	>32	2	>50	>64	>100	≥100
2-Propen-1-one, 1-(2,4-dihydroxy-6-methoxy-3,5-dimethylphenyl)-3-phenyl	8	Myrica serrata	65349-31-7	>32	8	25	>64	25	3.125
*O*-Methyllawsone	9	Swertia calycina	2348-82-5	16	8	12.5	>64	25	1.5625
Dihydrochelerythrine	10	Fagara zanthoxyloides	6880-91-7	8	>32	25	>64	25	3.125
Simplexene D	11	Swartzia simplex	New NP	>32	16	25	>64	>100	≥12.5
Waltherione G	12	Waltheria indica	1632043-42-5	>32	32	25	>64	ND	ND
Waltherione F	13	Waltheria indica	1632043-41-4	>32	8	12.5	>64	50	6.25
8-Deoxoantidesmone	14	Waltheria indica	NA	>32	16	25	>64	>100	12.5
Waltherione E	15	Waltheria indica	954367-81-8	>32	4	12.5	>64	>100	≥50
Pterostilbene	16	Sigma-Aldrich	537-42-8	32	32	50	>64	ND	ND
(5*S*,10*S*)-11,15-(*S*)-Dihydroxy-12-methoxyswartziarboreol G	17	Swartzia simplex	1830306-56-3	32	16	50	>64	50	3.125
Pulsatilla saponin D	18	Odontadenia puncticulosa	68027-15-6	>32	16	>50	>64	100	6.25
3β-*O*-[β-d-Xylopyranosyl-(1→3)]-α-l-rhamnopyranosyl-(1→2)-[β-d-glucopyranosyl- (1→4)]-α-l-arabinopyranosyl] hederagenin	19	Odontadenia puncticulosa	NA	>32	8	>50	>64	50	6.25
Garcinone C	20	Phytolab	76996-27-5	8	32	>50	>64	50	6.25
Pennogenin tetraglycoside	21	Phytolab	68124-04-9	4	8	>50	>64	6.25	1.5625
Tomatidine hydrochloride	22	Phytolab	6192-62-7	<1	16	>50	>64	>100	≥200
Formosanin C	23	Phytolab	50773-42-7	1	4	25	>64	12.5	12.5
Medicagenic acid	24	Phytolab	599-07-5	>32	2	>50	>64	>100	≥100
Pyridoxatin	25	Sigma-Aldrich	135529-30-5	4	32	25	16	100	25
Isograndifoliol	26	Perovskia atriplicifolia	1445475-53-5	16	32	>50	>64	100	25
Taxodion	27	Salvia leriifolia	19026-31-4	16	8	12.5	>64	50	3.125
Waltherione N	28	Waltheria indica	New NP	>32	32	12.5	>64	ND	ND
5-(*R*)-Vanessine	29	Waltheria indica	New NP	>32	32	12.5	>64	ND	ND
Waltherione Q	30	Waltheria indica	New NP	>32	32	25	>64	ND	ND
Antidesmone	31	Waltheria indica	222629-77-8	>32	32	12.5	>64	ND	ND
Waltherione I	32	Waltheria indica	1632043-44-7	>32	32	12.5	>64	ND	ND
Waltherione J	33	Waltheria indica	1632043-46-9	>32	16	12.5	>64	50	3.125

a CAS, Chemical Abstracts Service.

b The threshold for antifungal activity was 32 µg/ml.

c NA, not available.

d ND, not determined.

e The selectivity index was equal to the LD_50_/MIC for C. albicans.

**FIG 1:**
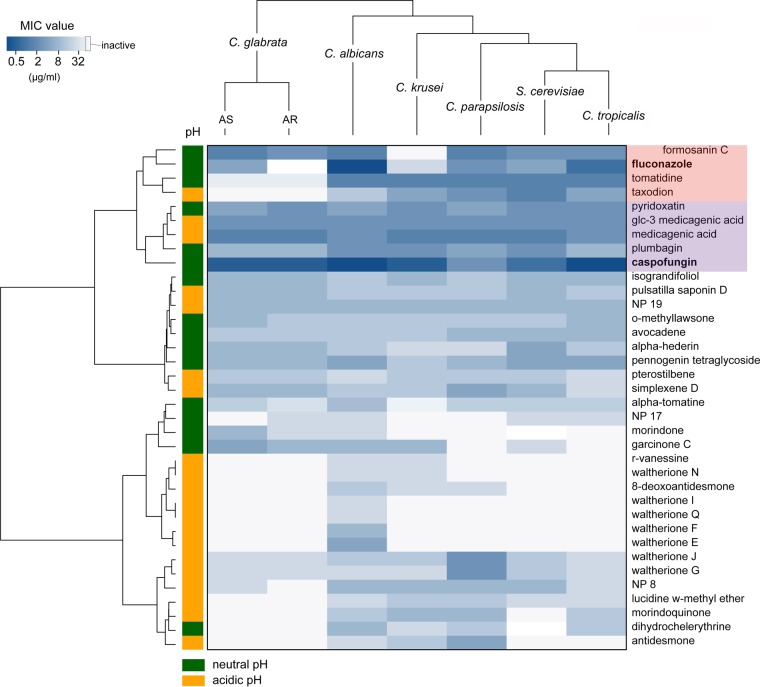
Heatmap and cluster analysis of the activity profiles of 33 active NPs. The MIC values of the NPs against seven yeast species were ordered by hierarchical clustering using the Euclidean distance method and are represented with a heatmap in which intensity is indicated by a color code (dark blue, MIC of 0.125 μg/ml; light blue, MIC of 32 μg/ml; white, MIC of >32 μg/ml [inactive]). The pH at which activity was detected is indicated by the color in the first column (orange, pH 4.6; green, pH 7). Fluconazole and caspofungin were added to the list of compounds, and their clusters are highlighted in red and purple, respectively.

In order to further characterize their antifungal properties, active NPs were profiled for their spectra of activity against several other clinically relevant Candida strains (C. glabrata, C. tropicalis, C. parapsilosis, and C. krusei) as well as another related nonpathogenic yeast (Saccharomyces cerevisiae). As shown in Fig. 1, a variety of activity profiles was observed, with some compounds being active against all strains, while others were active against only a small subset of species, suggesting a diversity in their modes of action and in the target cell response. C. glabrata strains (azole-sensitive [AS] and azole-resistant [AR] strains) exhibited the most resistant phenotypes. To identify compounds with high antifungal potential, two major clinical antifungal agents (fluconazole and caspofungin) were added to the group of NPs selected for testing, and all clustered according to their activity profiles. Four NPs (pyridoxatin, Glc-3-medicagenic acid [medicagenic acid 3-*O*-glucopyranoside], medicagenic acid, plumbagin) were grouped together with caspofungin (Fig. 1, purple highlight) and exhibited strong inhibitory activity against all species tested. The nearest neighboring cluster contained fluconazole and included three other NPs (formosanin C, tomatidine, and taxodion; Fig. 1, red highlight). This cluster was characterized by strong activity overall, but specific yeasts showed reduced susceptibility to the members of this cluster, such as C. *glabrata* (AR) and C. krusei strains, which showed reduced susceptibility to fluconazole, and C. glabrata strains, which showed reduced susceptibility to tomatidine.

Biofilms are defined as complex cell populations with intrinsic resistance to many antifungal drugs, whereas planktonic cells of the same organism do not show such intrinsic resistance (14). We tested the activities of selected NPs against C. albicans mature biofilms formed *in vitro* using an activity cutoff value higher than the one used for planktonic cells (MIC ≤ 50 μg/ml). Most of the compounds (25 out of 33) were active against biofilms and exhibited a MIC against biofilm cells higher than the MICs measured against planktonic cells, except for NPs originating from Waltheria indica. The antibiofilm activity of these NPs was previously reported (15).

We next tested the activity of the selected NPs (16) against a Gram-negative bacterium (Escherichia coli). All but one compound (pyridoxatin; Table 1) were inactive against this bacterial species (MIC > 64 μg/ml), arguing for fungus-specific inhibitory activity.

The last step in the screening process was to determine the potential acute toxicity of selected NPs on mammalian cells. The 24 most active antifungal NPs (MICs ≤ 16 μg/ml) were therefore tested by standard cytotoxicity assays on HeLa cells, and the 50% lethal dose (LD_50_) was determined. Our results indicated that only 3 NPs, tomatidine, medicagenic acid, and Glc-3 medicagenic acid, showed selectivity indices (SIs) with an acceptable range (>100). The results of all the antifungal profiling assays are summarized in Table 1. Since the antifungal activities of the two medicagenic acid-based NPs have already been reported (17), we focused our efforts on understanding the antifungal properties and mode of action of tomatidine.

### Antifungal activity of tomatidine.

**FIG 2:**
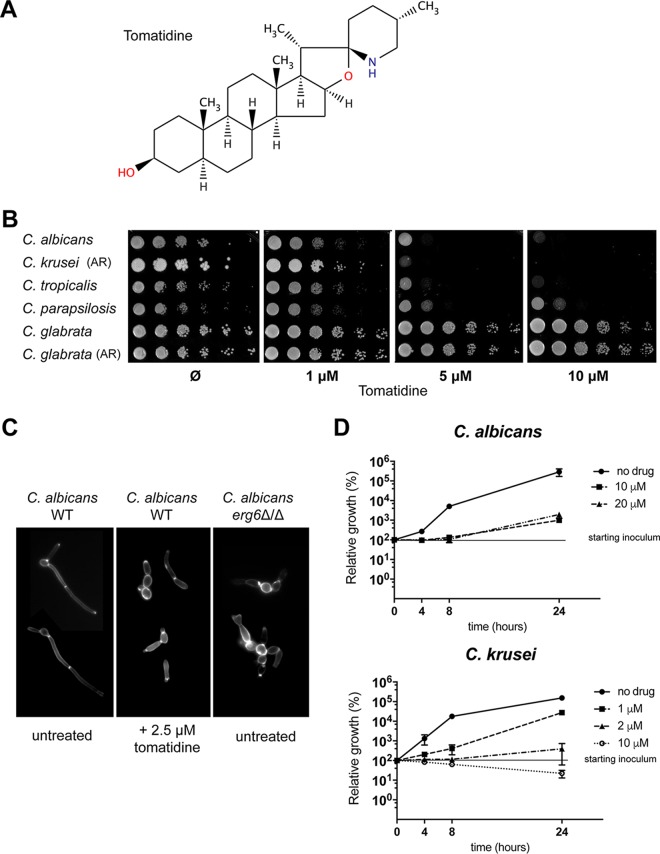
Tomatidine susceptibility assays with Candida spp. (A) Structure of tomatidine. (B) Spotting susceptibility assays with Candida spp. on YEPD agar plates. Tenfold serial dilutions of the indicated strains were spotted onto agar plates containing increasing tomatidine concentrations (0 [DMSO], 1, 5, or 10 μM tomatidine) and were incubated for 1 day at 34°C. AR, azole-resistant strain. (C) Calcofluor white staining of C. albicans cells exposed to tomatidine. Cells were exposed to 2.5 μM tomatidine for 3 h or left untreated (1% DMSO) in RPMI medium (0.2% glucose) at 37°C and then labeled with calcofluor white to stain the chitin. The cells depicted here are representative of the vast majority of cells from three independent experiments. Magnifications, ×100. (D) Time-kill assay for tomatidine-treated cells. C. albicans and C. krusei cells (2 ×10^5^) were treated with different supra-MICs of tomatidine in YEPD, and the numbers of CFU per milliliter were determined after 4, 8, and 24 h of exposure. The levels of growth (numbers of CFU per milliliter) relative to the initial level (at time zero, which was considered 100%) were calculated. Experiments were performed in duplicate, with average and SEM values being graphically represented. The *y* axis is on a log_10_ scale.

Tomatidine (Fig. 2A) is a sterol alkaloid produced by the tomato (Solanum lycopersicum). It is the precursor of the glycol sterol alkaloid α-tomatine, which is a well-known antifungal saponin with activity against phytopathogens (18). The antifungal and antiparasitic activities of tomatidine against S. cerevisiae and some parasites, such as Leishmania amazonensis and Phytomonas serpens, have been reported (19–21). These studies revealed that tomatidine exposure induces a perturbation of ergosterol biosynthesis and suggested that the C-24 sterol methyltransferase (24-SMT-Erg6) within the sterol biosynthetic pathway is a potential target. Erg6 is responsible for the key structural difference between cholesterol and ergosterol (21, 22). This is consistent with the fact that the *ERG6* gene is present in fungi but absent in higher eukaryotes, such as mammals. Erg6 therefore represents an attractive target for the development of antifungal agents.

The results of tomatidine susceptibility assays with several clinically relevant Candida species are shown on Fig. 2B. All strains, except C. glabrata, were highly susceptible to tomatidine. Similar results were obtained by standard microdilution susceptibility assays, with C. krusei and C. albicans showing the highest sensitivities (MIC = 0.625 μM) (Table 2). We used a larger panel of C. albicans isolates (*n* = 9) and found that the tomatidine MIC_50_ was 0.3125 μM. MIC_50_ values for both C. krusei (*n* = 8) and C. tropicalis (*n* = 9) were 1.25 μM (Table S4). Tomatidine was not active against C. albicans mature biofilms (Table 1), but the ability of C. albicans to form hyphae under filamentation-inducing *in vitro* conditions was severely compromised in the presence of tomatidine. As shown in Fig. 2C, the addition of tomatidine to C. albicans cultures under hypha-inducing conditions resulted in the formation of pseudohyphae and/or unseparated budding yeast cells. Tomatidine exhibited fungistatic activity against C. albicans, as revealed by time-kill assays performed with different drug concentrations (Fig. 2D). Tomatidine (10 μM) was observed to have only weak fungicidal activity against C. krusei, with the number of CFU being decreased by about 80% compared to that in the starting inoculum after 24 h of incubation (Fig. 2D).

**TABLE 2:** Tomatidine and fluconazole MICs for Candida spp.

Strain	MIC^*b*^					
	RPMI (pH 7)		YNB (pH 7)		YPED (pH 6.5)	
	Tomatidine	Fluconazole	Tomatidine	Fluconazole	Tomatidine	Fluconazole
C. albicans (SC5314)	0.625	0.125	0.625	0.5	1.25–2.5	0.5
C. krusei (DSY471) (AR^*a*^)	0.625	32	0.625	32	0.3125	>128
C. tropicalis (DSY472)	1.25	0.25–0.5	5	>128	2.5	32
C. parapsilosis (DSY473)	10	2	2.5	8	5	16
C. glabrata (DSY562)	>40	2	>40	32	>40	64
C. glabrata (DSY562) (AR)	>40	128	>40	>128	>40	>128

a AR, azole resistant.

b MICs are in micromolar for tomatidine and micrograms per milliliter for fluconazole.

### Global transcriptional analysis of tomatidine-treated C. albicans cells.

**FIG 3:**
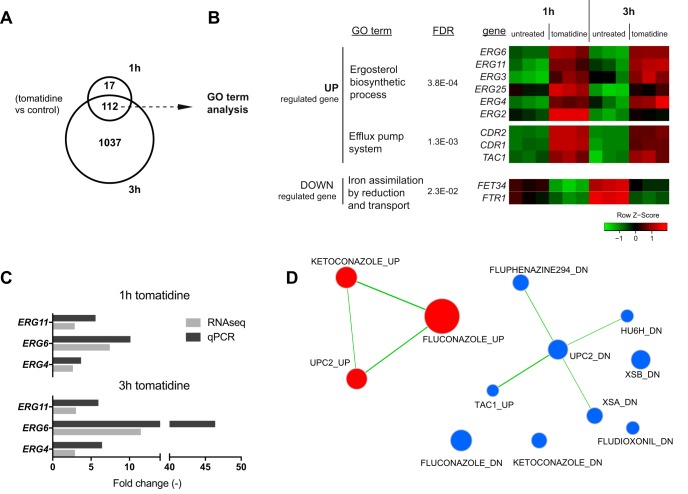
Genome-wide transcriptional analysis of tomatidine-treated cells. (A) Venn diagram showing the number of genes differentially regulated by tomatidine (2.5 μM) compared to their regulation in control cells after 1 and 3 h of exposure (FDR > 0.05, fold change ≥ 2). (B) GO term analysis of the commonly up- and downregulated genes upon 1 h and 3 h of tomatidine treatment. The GO terms and their FDR values are indicated (left), and expression of the corresponding genes is represented with a heatmap for both drug exposure durations (right). The scaled expression of each gene, denoted as the row Z-score, is plotted on a red-green color scale, with red indicating a high level of expression and green indicating a low level of expression. A Z-score of 0 (black) corresponds to the mean expression level of a particular gene, and the Z-score scale indicates the numbers of standard deviations (positive or negative). Genes within each GO term group are listed according to an increasing adjusted *P* value. (C) qPCR analysis of *ERG11*, *ERG6*, and *ERG4* expression in tomatidine-treated cells. The increase in the fold change in expression compared to the level of expression in untreated cells is represented as a bar plot. A *t* test analysis of the relative level of gene expression between the treated and untreated groups was performed for each gene, and significant differences were detected (*P* values for all comparison, <0.05). (D) Gene set enrichment analysis (GSEA) of C. albicans genes regulated by tomatidine. The list of drug-regulated genes was generated from published transcriptional data (see Data Set S2 in the supplemental material; data file name, Candida_drug_treatment.gmt). Tomatidine-regulated genes (3 h) are ranked according to their fold change in expression. The list was then imported into GSEA software. Analysis parameters were as follows: normalization mode (norm), meandiv; scoring scheme parameter, weighted; minimal gene set size, 15; number of permutations, 1,000; maximal gene set size, 500. GSEA results were uploaded into Cytoscape (version 3.0) software with the following parameters: *P* value cutoff, 0.01; FDR *q* value, 0.05. Red nodes, enriched genes among upregulated genes from GSEA; blue nodes, enriched genes among downregulated genes from GSEA. Nodes (colored circles) are connected by edges (connecting lines) when overlaps exist between nodes. The size of the nodes reflects the total number of genes that are connected by edges to neighboring nodes. The labels of the list (corresponding to the list of genes upregulated [UP] and downregulated [DOWN] by drug treatments, gene deletions, or stress conditions) are indicated next to the nodes (see Data Set S2 for details). XSA and XSB, oxidative shock; HU6H, hydroxyurea treatment.

Genome-wide transcriptional analysis in the presence of given drugs has often been used as a means to highlight their modes of action and to propose possible cellular targets (23). The effect of tomatidine treatments on the C. albicans transcriptome was investigated *in vitro* using two different exposure times (1 and 3 h) and a fixed drug concentration (2.5 μM). This drug concentration corresponds to the MIC obtained in 1% yeast extract, 2% peptone, and 2% glucose (YEPD) medium (Table 2) and led to 30% and 50% growth inhibition after 1 h and 3 h of incubation, respectively (data not shown). Total RNA was recovered from treated and untreated cells, and genome-wide transcriptional analysis was performed using transcriptome sequencing (RNA-seq) (see Materials and Methods). After 1 and 3 h of tomatidine exposure, 129 and 1,149 genes (2% and 21% of the genes expressed by treated and untreated cells, respectively) were identified to be differentially expressed between treated and untreated cells (false discovery rate [FDR], ≤0.05; fold change, ≥2), respectively. One hundred twelve genes were found to be affected after both 1 and 3 h of exposure. Seventy-seven genes were upregulated, 32 genes were downregulated, and 3 genes were inversely regulated compared to their regulation under untreated conditions (Fig. 3A; Data Set S1). Gene Ontology (GO) term analysis of the 77 commonly upregulated genes revealed a clear alteration of the expression of 6 genes (*ERG2*, *ERG3*, *ERG4*, *ERG6*, *ERG11*, *ERG25*) involved in the ergosterol biosynthetic pathway (corrected *P* value < 0.0004) (Fig. 3B). Interestingly, other components of the ergosterol biosynthetic pathway were found to be upregulated by tomatidine after 1 h of exposure, including *ERG1*, *ERG5*, *ERG24*, and *UPC2* (the master sterol transcriptional regulator), and 3 h of exposure, including *ERG9* and *ERG28* (Data Set S1). Interestingly, among all the *ERG* genes differentially regulated by tomatidine exposure, the most affected was *ERG6*, which showed 7.4- and 11.8-fold changes in the level of expression after 1 and 3 h of treatment, respectively. This important fold change in expression was confirmed by quantitative PCR (qPCR) analysis (up to a 40-fold increase at 3 h of treatment) (Fig. 3C).

Response to steroid hormone stimulus was another overrepresented GO term among the upregulated genes (corrected *P* value < 0.002). The three corresponding genes were part of the *TAC1* regulon and are involved in the major drug efflux system in C. albicans: the two ABC transporters (*CDR1* and *CDR2*) and the transcriptional regulator itself (*TAC1*) (Fig. 3B). qPCR analysis of *ERG4*, *ERG6*, *ERG11*, *CDR1*, and *CDR2* expression from the same RNA samples validated the results of RNA-seq data analysis (Fig. 3C and S1).

GO analysis identified only one enriched GO term (ion transport; corrected *P* value < 0.003) for commonly downregulated genes (32 genes), with 2 genes being involved in iron or calcium transport (*FET34*, *FTR1*).

Exposure of C. albicans cells to tomatidine for 3 h led to large transcriptional changes (in a total of 1,149 genes), with 1,037 genes specifically being affected after that exposure time. GO term analysis identified processes that are characteristics of growth inhibition/arrest or drug-related stress, such as glucose transport in upregulated genes and the transcriptional and translational machinery in downregulated genes (Data Set S1).

In order to better characterize the response to tomatidine, we used gene set enrichment analysis (GSEA) to identify transcriptional signatures from other drug-induced transcriptional studies overlapping the present tomatidine transcriptional data (Data Set S2). As shown in Fig. 3D, genes up- and downregulated by tomatidine (red and blue, respectively) shared the highest number of regulated genes with the fluconazole_UP/DOWN gene sets, followed by another azole-related gene set (ketoconazole_UP/DOWN) and the gene set from cells lacking the master regulator of ergosterol genes (*UPC2*). Using another set of previously published transcriptional data obtained under several growth conditions inducing stress *in vitro* (such as hypoxia [24]), the set of genes regulated under tomatidine treatment conditions shared genes regulated under hypoxic conditions, in which the adaptive response is mainly mediated by *UPC2* (Fig. S2) (25). Taken together, these analyses reveal that tomatidine has a transcriptional signature closely related to that of azole drugs and strongly suggest that the ergosterol biosynthetic pathway is also targeted by this NP.

### Tomatidine as an inhibitor of the sterol biosynthetic pathway.

As suggested by the results of our transcriptional analysis, tomatidine is likely to interfere with ergosterol biosynthesis, given that the upregulation of *ERG* genes upon treatment has been shown to be a mechanism in the cell involved in the response to drugs targeting the ergosterol pathway (26). In order to detect more precisely the step(s) of this pathway that was affected, we performed an in-depth analysis of the sterol content of C. albicans, C. krusei, and S. cerevisiae cells treated with tomatidine. Total sterols were extracted from yeast cells and next subjected to gas-liquid chromatography coupled with mass spectrometry (GLC-MS) analysis.

**FIG 4:**
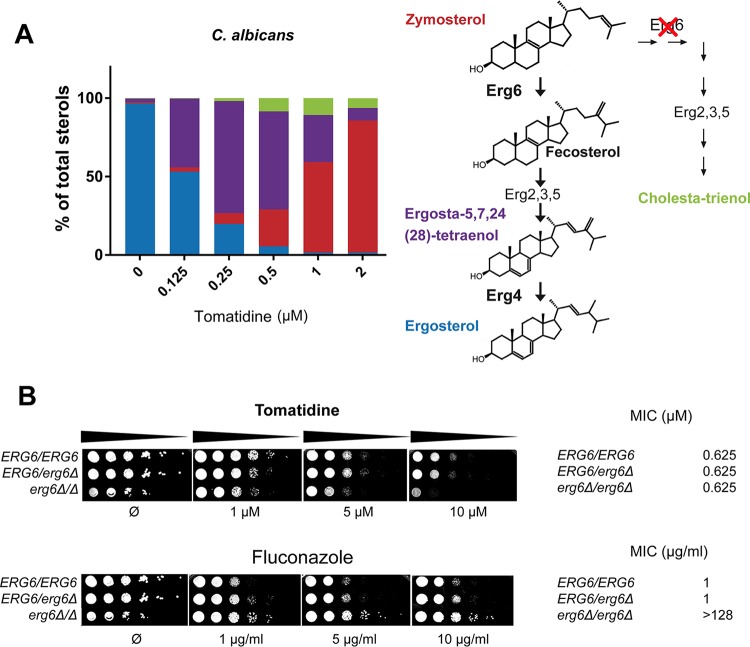
Analysis of total sterols in tomatidine-treated cells by GLC-MS. (A) Sterol composition of C. albicans cells treated with increasing amounts of tomatidine. (Left) The results of GLC-MS analysis of cells exposed to tomatidine indicate the percentages of the different sterols in a bar plot. The bar colors correspond to those of the sterol molecules illustrated on the right, where the last steps in the ergosterol biosynthetic pathway and an alternative pathway following *ERG6* inhibition are shown. The experiment was repeated in duplicate and gave similar results each time. (B) Assay of the susceptibility of the C. albicans *ERG6* deletion strain. Wild-type (*ERG6*/*ERG6*), heterozygous (*ERG6*/*erg6*Δ), or homozygous *ERG6* (*erg6*Δ/*erg6*Δ) deletion strains were subjected to a serial dilution susceptibility assay on YEPD plates containing the indicated concentration of tomatidine or fluconazole (left) and to standard MIC determination assay in YNB (pH 7) medium (right).

As shown in Fig. 4A, untreated C. albicans cells contained, as expected, ergosterol as the most abundant sterol (>96% of total sterols). Zymosterol (the product known to accumulate in the *ERG6* [encoding C-24 sterol methyltransferase] mutant) was the major sterol (84% of total sterols) in tomatidine-treated cells, thus suggesting strong inhibition of this enzymatic step by tomatidine. Furthermore, careful analysis of the sterol content allowed the identification of a small amount of two other sterol intermediates, including cholesta-trienol (6%) and ergosta-5,7,24(28)-tetraenol (8%). Enzymes involved in the late stage of ergosterol biosynthesis usually act independently of each other, which enables the pathway to proceed even if one step is inhibited by a drug or is genetically suppressed, leading to the synthesis of alternative sterols. One of those is cholesta-trienol, which was detected in tomatidine-inhibited cells, where the successive actions of Erg2, Erg3, and Erg5 occurred in the absence of previous Erg6 activity. However, Erg4, the last enzyme involved in the sterol pathway, first requires the action of Erg6 to produce its substrate. More specifically, the methylene group added by Erg6 at position C-24 of zymosterol is the substrate for the C-24 reductase activity of Erg4. Therefore, detection of the Erg4 substrate [ergosta-5,7,24(28)-tetraenol] was unanticipated and suggested that (i) tomatidine also inhibited Erg4 and (ii) Erg6 was not completely inhibited by tomatidine at the concentration tested.

In order to be able to assess more accurately the inhibition of Erg4 activity, we next performed a progressive decrease of Erg6 inhibition by titrating down the tomatidine concentration. As shown in Fig. 4A, a decrease in the tomatidine concentration from 2 μM to 0.25 μM resulted in the release of Erg6 inhibition (the amount of zymosterol as a proportion of total sterols decreased from 84% to 7%) and the accumulation of ergosta-5,7,24(28)-tetraenol (which consisted of up to 72% of total sterols with 0.25 μM tomatidine), thus confirming the ability of tomatidine to inhibit Erg4. As the concentration of tomatidine required to accumulate zymosterol and ergosta-5,7,24(28)-tetraenol as a proportion of total sterols of up to 84% and 72%, respectively, is approximately 2 μM and 0.25 μM, respectively, one can estimate that tomatidine is about 10 times more effective as an inhibitor of Erg4 than as an inhibitor of Erg6. Interestingly, this Erg4/Erg6 dual inhibition was also found in C. krusei but not in S. cerevisiae, in which only Erg6 inhibition could be detected (Fig. S3).

Following this analysis, a C. albicans *ERG6* deletion strain was engineered in order to validate that Erg6 is a target of tomatidine. We reasoned that if the Erg6 target is absent, then tomatidine will not be active against this mutant. As already described, the deletion of *ERG6* results in increased susceptibility to a large variety of drugs, with the exception of azoles and polyenes (27). The *erg6*Δ/*erg6*Δ mutant was indeed resistant to fluconazole, with the MIC for the mutant being 128-fold higher than that for the wild type (Fig. 4B). Interestingly, the *erg6*Δ/*erg6*Δ mutant constructed here was as susceptible to tomatidine as the parental strain (Fig. 4B). This indicates a pleiotropic susceptibility of the strain or a potential secondary target(s) of tomatidine, in addition to the ergosterol pathway. Similar results were obtained using an S. cerevisiae *ERG6* mutant (Fig. S4).

### Use of a forward genetic approach to identify targets of tomatidine.

**FIG 5:**
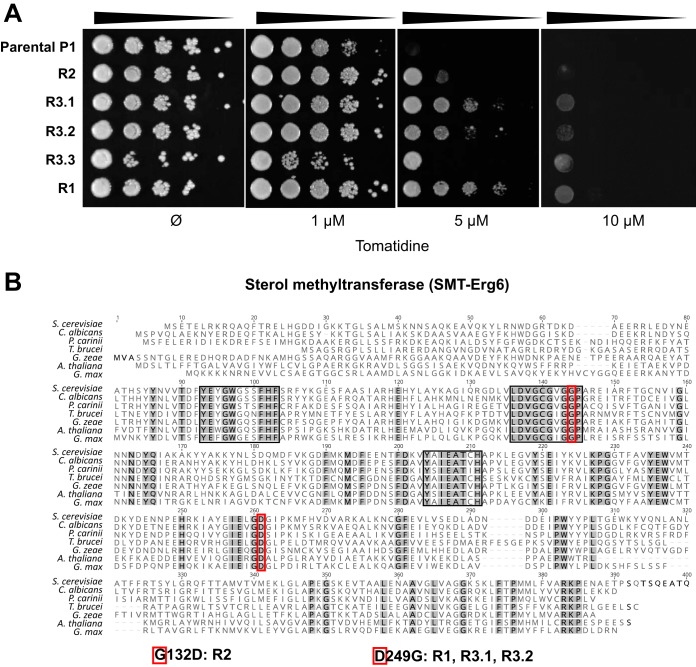
Forward genetic approach in S. cerevisiae. (A) Tomatidine susceptibility of resistant mutants. Fungal cells were spotted on YEPD agar containing different concentrations of tomatidine (and a drug-free control), as indicated. The plates were incubated at 30°C for 48 h. (B) Alignment of the C-24 sterol methyltransferase (SMT) protein sequences from distant eukaryotic species (fungi, S. cerevisiae, C. albicans, Pneumocystis carinii, and Giberella zeae; green plants, Glycine max and Arabidopsis thaliana; euglenozoan, Trypanosoma brucei). Gray, conserved residues; black boxes, highly conserved regions that have a role in substrate binding and enzymatic activity (30); red boxes, the two mutated residues found in C-24 sterol methyltransferase (Erg6) of tomatidine-resistant strains (G132D and D249G) conserved across all aligned sequences. Strains carrying the *ERG6* mutation are indicated below the alignment. The GenBank accession numbers of the protein sequences are as follows: S. cerevisiae, KZV08836; C. albicans, AOW28252; P. carinii, KTW25893; *G. zeae*, ESU10532; *G. max*, NP_001238391; A. thaliana, NP_173458; and T. brucei, AAZ40214. A multiple-sequence alignment was performed using the MUSCLE program in Geneious software (version 9.1.4; default parameters).

A forward genetic screen in S. cerevisiae was finally undertaken as an unbiased way to discover the target(s) of tomatidine. The strain chosen in this approach lacked *PDR5* and *MSH2* to avoid multidrug transporter-dependent resistance mechanisms and to increase the rates at which resistance mutations may occur, respectively (28, 29). A *pdr5*Δ *msh2*Δ strain (strain P1) was plated on YEPD medium containing 10 μM tomatidine, and after a screening of more than 2 × 10^8^ cells, one resistant mutant (mutant R1) was isolated. To obtain additional resistant mutants, a second strategy inspired by Ojini and Gammie (29) was developed and consisted of submitting cells to two drug exposure periods in liquid medium (72 and 48 h) interspersed by a period of drug-free growth (48 h). Cultures with robust growth at the end of the experiment were further plated on solid YEPD medium containing 10 μM tomatidine, which resulted in the isolation of four resistant mutants (mutants R2, R3.1, R3.2, and R3.4) from two different cultures. The tomatidine resistance of the five mutants obtained was confirmed by a serial dilution assay (Fig. 5A). MIC results showed a 4-fold decrease in susceptibility for R1, R3.1, and R3.2 compared to that of parental strain P1. Isolates R3.3 and R2 exhibited only a slight decrease in susceptibility. Tomatidine resistance was specific, since the results of susceptibility assays with drugs of different classes (fluconazole, caspofungin, and amphotericin B) showed that the MICs of fluconazole, caspofungin, and amphotericin B for the mutant strains were in the range of those for strain P1 (Table 3). Simplified sterol composition analysis using the unique spectrophotometric absorbance signature of sterols confirmed that the mutants had a normal sterol composition with no detectable differences from that of the parental strain (Fig. S5A).

**TABLE 3:** MICs of different antifungals for S. cerevisiae strains

Strain	MIC^*a*^			
	Tomatidine	Fluconazole	Caspofungin	Amphotericin B
P1	1.25	4	0.25	2
R2	1.25	4	0.25	2
R3.1	5	4	0.25	2
R3.2	5	4	0.25	2
R3.3	2.5	4	0.25	2
R1	5	4	0.25	2
IMX581	5	16	0.125	2
D249G	20	16	0.125	2
G132D	20	4	0.125	2

a MICs are in micromolar for tomatidine and micrograms per milliliter for fluconazole, caspofungin, and amphotericin B.

The five mutant genomes were sequenced, and the sequences were compared with the sequence of parental strain P1. Alignment of the sequences of the parental and mutant genomes with the sequence of the reference genome (S288C) was followed by the identification of nonsynonymous polymorphisms uniquely present in the coding regions of the resistant mutants (see Materials and Methods). All the nonsynonymous mutations inventoried in the five resistant mutants (between 26 and 60, depending on the strain) are listed in Data Set S3. Interestingly, four out of the five resistant mutants contained a missense mutation in *ERG6* with the following amino acid substitutions: D249G for R1, R3.1, and R3.2 and G132D for R2. These amino acid residues might play an important role in Erg6 function, as they are conserved among fungi, plants, and protozoa (30) (Fig. 5B), but they seemed dispensable for normal ergosterol synthesis. To determine if the two mutations were sufficient to confer tomatidine resistance, the G132D and D249G substitutions were inserted in a wild-type S. cerevisiae strain (IMX585) using the site-directed clustered regularly interspaced short palindromic repeat (CRISPR)-Cas9 genome editing technology (31). Both strains (the G132D and D249G mutants) were 4-fold more resistant to tomatidine than the wild type (Table 3), thus recapitulating the tomatidine-resistant phenotype of the original resistant mutants (or, for the G132D mutant, increasing its level of resistance). Analysis of the sterol profiles showed that the G132D strain, in contrast to the corresponding original resistant mutant and the D249G strain, exhibited an altered sterol composition with profiles intermediate between those of an *ERG6* deletion mutant and a wild-type strain (Fig. S5B). This might explain the increase in fluconazole susceptibility observed for this strain (Table 3).

While the last resistant mutant (mutant R3.3) retained a wild-type allele of *ERG6*, it exhibited a frameshift mutation in *ACE2*. This gene encodes a transcription factor required for septum degradation after cytokinesis (32). Strain R3.3 exhibited a multicellular clumping phenotype, which was identical to that of an *ACE2* deletion strain (Fig. S6). A decrease in tomatidine susceptibility was observed in the *ACE2* deletion strain and thus recapitulated the resistance phenotype of the initial R3.3 strain (Table 3).

### Tomatidine is targeted by efflux pumps.

**FIG 6:**
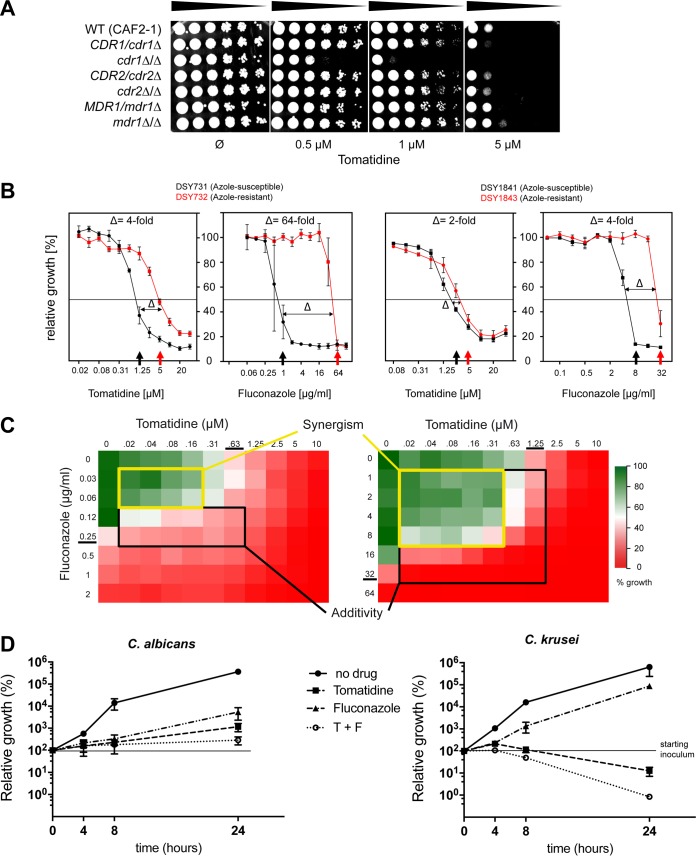
Tomatidine targets efflux pumps and induces only partial cross-resistance with fluconazole. (A) Serial dilution susceptibility assays of C. albicans strains carrying a deletion of efflux pump genes (the genotypes are indicated) on YEPD agar plates. Tenfold serial dilutions of the indicated strains were spotted onto agar plates containing increasing tomatidine concentrations (0, 0.5, 1, 5 μM) and were incubated for 1 day at 34°C. (B) Tomatidine MICs of two matched pairs of azole-susceptible (AS) and azole-resistant (AR) strains. Determination of the MICs of tomatidine and fluconazole was performed in YNB medium, and the MICs are indicated by the arrows. Δ, difference in the fold change in the MICs between matched AS and AR strains. (C) Two heatmaps representing color plots of the results of checkerboard MIC tests. Each box corresponds to the relative growth (compared to that of the drug-free control) resulting from a specific combination of tomatidine and fluconazole (RPMI medium, pH 7). The MIC of each drug is underlined. Zones with a black border, additive interaction (FIC index, between 2 and 0.5); zones with a yellow border, synergistic effect (FIC, <0.5). (D) Time-kill assay of C. albicans and C. krusei cells exposed to a combination of tomatidine (T) and fluconazole (F). Cells were treated with the indicated amounts of the drugs, individually or in combination, in YEPD medium; the number of CFU per milliliter was determined after 4, 8, and 24 h of exposure; and the number of CFU relative to that at time zero (for which the number of CFU was considered 100%) was calculated. Experiments were performed in duplicate, with average and SEM values being graphically represented. The *y* axes are on a log_10_ scale. On the graph for C. krusei, a dashed line was plotted at *y* equal to 1 to delimit the fungicidal threshold (2 times a log_10_ decrease).

The forward genetic approach was designed to exclude multidrug transporter-dependent resistance mechanisms by using an S. cerevisiae strain lacking *PDR5*. However, the activation of drug efflux in yeast is a common cell defense mechanism against toxic drugs, as observed in our C. albicans genome-wide transcriptional analysis, in which the genes of the *TAC1* regulon (including *CDR1* and *CDR2*) were among the genes upregulated by exposure to tomatidine (Fig. 3B). To address whether tomatidine was a target of the efflux pump system in C. albicans, a set of deletion mutants lacking *CDR1*, *CDR2*, or *MDR1* (another import efflux pump belonging to the major facilitator superfamily of transporters) was used to evaluate their tomatidine susceptibility. The *cdr1*Δ/*cdr1*Δ strain was the only one to exhibit increased susceptibility to tomatidine; the *CDR2* and *MDR1* mutants and the wild-type strain did not (Fig. 6A). These data clearly indicate that, like many other drugs, such as fluconazole (33), tomatidine is targeted by the *CDR1* efflux pump.

A major problem arising during clinical treatment of candidiasis is the emergence of resistant isolates. One important mechanism underlying the development of resistance consists of the upregulation of efflux pumps by the acquisition of hyperactive alleles of their regulators (16). To evaluate the potential cross-resistance between fluconazole and tomatidine of strains with hyperactive *TAC1* alleles, we measured the tomatidine susceptibility of two pairs of matched azole-susceptible (AS) and azole-resistant (AR) C. albicans clinical isolates. The AR isolates carried hyperactive *TAC1* alleles. While the difference in the fluconazole MIC between strains DSY732 (AR) and DSY731 (AS) was 64-fold, the tomatidine MIC diverged by only 4-fold between the two strains (Fig. 6B, left; Table 4). Similar results were obtained using C. albicans clinical isolates DSY1843 (AR) and DSY1841 (AS). Azole and tomatidine MICs increased by only 4- and 2-fold, respectively (Fig. 6B, right; Table 4). Taken together, these results suggest that the common mechanism of resistance to azoles triggered by *TAC1* hyperactivity seems to have a limited effect on tomatidine susceptibility.

**TABLE 4:** Susceptibility of C. albicans clinical isolates to tomatidine and fluconazole

Strain^*a*^	MIC^*b*^	
	Tomatidine	Fluconazole
DSY731 (AS)	0.625	1
DSY732 (AR)	2.5	64
DSY1841 (AS)	2.5	8
DSY1843 (AR)	5	32

a AR, azole resistant; AS, azole susceptible.

b MICs were determined in YNB (pH 7) and are in micromolar for tomatidine and micrograms per milliliter for fluconazole.

As C. albicans exhibited limited cross-resistance between fluconazole and tomatidine, we next investigated the potential synergistic effect of their combination. Classical checkerboard combination assays were performed, and fractional inhibitory concentration (FIC) values were determined (as described in Materials and Methods). As expected for drugs targeting the same pathway, fluconazole and tomatidine exhibited a strong additive effect in both C. albicans and C. krusei, with cell growth inhibition by the two drugs in combination being more than 50%, which is in the zone of additivity (Fig. 6C). We then tested if the combination of the two drugs changed their fungistatic properties. Interestingly, a time-kill assay showed that use of the fluconazole-tomatidine combination led to fungicidal activity against C. krusei (a >100-fold decrease in cells counts after 24 h) but not against C. albicans (Fig. 6D).

### *In vivo* activity of tomatidine.

**FIG 7:**
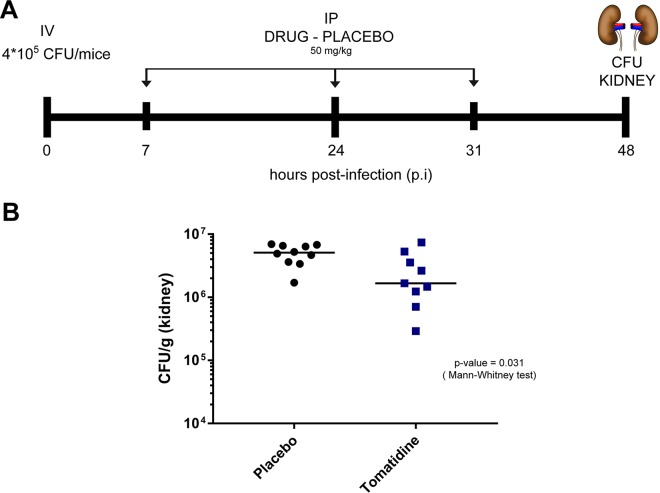
*In vivo* efficacy of tomatidine in a mouse model of systemic infection. (A) Schematic representation of the infection and treatment protocol. Mice were infected with 4 × 10^5^ CFU through the tail vein. Tomatidine treatment (50 mg/kg) (or placebo) was administered intraperitoneally at 7, 24, and 31 h postinfection (p.i.). At 48 h p.i., animals were sacrificed and kidneys were collected for determination of the number of CFU. (B) Fungal burden in tomatidine-treated mice. The number of CFU per gram of kidney is plotted for placebo- and tomatidine-treated mice. Significant differences in the distribution of the number of CFU were assessed using the Mann-Whitney test (*n* = 9 and 10 for the tomatidine- and placebo-treated mice, respectively; *P* = 0.031).

To confirm the strong potential for tomatidine to be a therapeutic agent, the *in vivo* efficacy of the compound was tested in a mouse model of C. albicans systemic infection. Due to its hydrophobic nature, a nanoparticle-based formulation of tomatidine was developed to allow its administration and to potentially enhance its bioavailability. Mice were infected through the tail vein with a C. albicans inoculum and were treated intraperitoneally (i.p.) with tomatidine (50 mg/kg of body weight) or placebo at 6 h, 24 h, and 31 h postinfection (p.i.). The numbers of CFU in the kidneys were then determined at 48 h p.i. As illustrated in Fig. 7, mice treated with tomatidine exhibited statistically significantly reduced numbers of CFU compared to the controls (Mann-Whitney test, *P* = 0.031), thus highlighting the *in vivo* activity of tomatidine and its therapeutic potential.

## DISCUSSION

### NPs as sources of antifungal agents.

The aim of our study was to identify promising natural compounds with antifungal activity, starting with a small-scale screening of carefully selected compounds, proceeding with an extensive *in vitro* characterization of their antifungal properties and cytotoxicity, and then extending to the identification of their cellular targets and the validation of their *in vivo* therapeutic potential.

A library of 199 natural products was built using both direct isolation from extracts of plants with documented antifungal properties (29 different plants were investigated, and 53% of the NPs investigated were isolated from these plants) and commercial acquisition after selection on the basis of structural similarities with known antifungals. The efficiency of the preselection process explains the high positive hit rate (17%; 33/199) of NPs with activity against human pathogens when a cutoff MIC value of ≤32 μg/ml was used. From an extended bioactivity profiling procedure, including assays on different fungal strains, a bacterial strain, and cytotoxicity assays for therapeutic index evaluation, only three interesting leads were identified. Tomatidine stood out as a novel anti-Candida drug with a putative promising target.

Tomatidine is a sterol alkaloid from tomato plants with a cholesterol-derived hydrophobic 27-carbon skeleton and serves as a precursor intermediate in the synthesis of a plant defense metabolite, the glycol sterol alkaloid α-tomatine (34). The latter has been characterized as an antifungal agent with activity against a large variety of phytopathogens and possesses membrane disruption properties (unspecific toxicity), caused by its ability to form complexes with cholesterol and ergosterol (18, 35). α-Tomatine is present in most green parts of tomato plants, where it is subjected to degradation during maturation. In contrast, tomatidine, the aglycone of α-tomatine, has a different mode of action, as it does not show any sterol binding activity (35). It is poorly active against phytopathogens, it exhibits no toxicity *in vivo*, and it is present in only trace amounts in the tomato plant (18, 19, 36). Tomatidine has been reported to have antifungal and antiparasitic activity against S. cerevisiae and Leishmania amazonensis (19, 20), and a putative target, the C-24 sterol methyltransferase (24-SMT-Erg6) was proposed. In the present study, the antifungal activity of tomatidine against important fungal pathogens was characterized in-depth for the first time. All Candida spp. except C. glabrata were susceptible to tomatidine (MIC = 0.25 to 10 μM; Table 2).

### Tomatidine mode of action.

Multilevel investigations were then conducted in order to determine its mode of action and to identify its molecular target. A first genomic strategy characterized a tomatidine-specific transcriptional signature in C. albicans and identified an important upregulation of the ergosterol biosynthetic genes, including *ERG6*, which was the most affected gene (Fig. 3). These results confirmed the findings of published studies of S. cerevisiae (19) and pointed out that the ergosterol pathway is the target of tomatidine. Interestingly, GSEA identified fluconazole to be a mediator of the transcriptional signature most closely related to that mediated by tomatidine (Fig. 3), which is consistent with their inhibitory activities being directed to the same pathway. Furthermore, this convergence of azole and tomatidine activity was also shown by microscopic analysis of the cytological effect of the drugs on the ultrastructure of C. albicans cells (see Fig. S7 in the supplemental material).

Considering the findings presented above, a detailed analysis of the sterols in C. albicans and C. krusei using GLC-MS resulted in the indirect identification of (i) Erg6 inhibition through a high level of accumulation of the Erg6 substrate zymosterol in cells treated with a high concentration of tomatidine and (ii) Erg4 inhibition through a high level of accumulation of ergosta-5,7,24(28)-tetraenol, its substrate, in cells treated with a low concentration of tomatidine (Fig. 4). As Erg4 needs Erg6 activity to obtain its substrate, the identification of this dual Erg4-Erg6 inhibition was made possible by the likely higher affinity of tomatidine for Erg4 than for Erg6. Both enzymes target the C-24 position in the ergosterol precursor and thereby probably exhibit a similar binding pocket conformation, even if their protein sequence identity is low (15%). A dual effect on Erg4 and Erg6 had already been reported in S. cerevisiae for azasterol, a sterol carrying a nitrogen in the side chain (37), and thus reinforces the idea of structural similarities between the targets. Dual inhibition by tomatidine was not detected in S. cerevisiae, where no ergosta-5,7,24(28)-tetraenol was identified, even at a low concentration. This suggests that either Erg4 in S. cerevisiae is not inhibited by tomatidine or it has a lower affinity for tomatidine than Erg6 does. These intraspecies differences could be attributed to Erg4-independent genetic evolution, as single point mutations in *ERG6* can affect sensitivity to tomatidine.

It has been shown that deletion of the target of fluconazole (*ERG11*) in C. albicans caused a marked increase in its resistance to the drug (38). We applied the same paradigm to tomatidine and tested the susceptibility of an *ERG6* deletion strain. Late-acting ergosterol genes are not essential for cell viability, and ergosterol is replaced by altered sterols in the membrane of deletion strains (39). However, these substitutions impact the regulation of membrane permeability and fluidity and are associated with diverse phenotypic alterations. A yeast *ERG6* deletion mutant showed pleiotropic hypersensitivity to a broad range of antifungal compounds and metabolic inhibitors, reflecting increased membrane permeability and passive diffusion by small molecules (mostly hydrophobic) (27, 39, 40). The fact that the tomatidine susceptibility of the C. albicans *ERG6* deletion strain was similar to that of the wild-type strain was intriguing. It is known that *ERG6* deletion can alter the permeability of the cell membrane to different drugs, and this phenotype has been utilized by others in order to improve the effects of different drugs (41). Given that *ERG6* deletion results in a slightly increased susceptibility to tomatidine compared to that of the wild type, it suggests that the compound could still target other cellular components. Consistent with this hypothesis, we identified *ACE2* to be another putative target of tomatidine (see below). It is interesting to note that the C. albicans *ERG6* deletion strain was resistant to fluconazole (Fig. 4B), which indicates drug-dependent susceptibility phenotypes and thus excludes the possibility of an unspecific hypersensitivity response to a given stressor.

The aim of our use of a forward genetic approach in S. cerevisiae was to identify alternative targets of tomatidine by avoiding the interference of efflux pump hyperactivity mechanisms. However, our screen revealed Erg6 to be a major target, and several explanations can be proposed for this. First, the set of resistant mutants that we obtained was relatively small (5), thus limiting the ability to discover additional targets. Second, an alternative target could be accessible or present only in an *ERG6* deletion background, in which altered sterols with compromised cell membrane functions are present. The hypothesis of a pleiotropic effect of the *ERG6* deletion on susceptibility to small hydrophobic molecules was tested using a sterol alkaloid (solasodine) which possesses a chemical structure highly similar to that of tomatidine (they differ only in the planar configuration of the piperidine ring and an unsaturated alpha ring bond). While this molecule was inactive against a C. albicans wild-type strain (MIC > 40 μM), the deletion of *ERG6* increased drug susceptibility (MIC = 20 μM) and thus argued for an unspecific drug hypersensitivity of this strain. Furthermore, *in vitro* filamentation studies with C. albicans (Fig. 2D) attested to the similar pseudohyphae and/or unseparated budding yeast cell phenotype between tomatidine-treated wild-type cells and cells depleted *of ERG6*, arguing for the possibility that tomatidine has a major target. The forward genetic approach identified *ACE2* to be a possible tomatidine target. Interestingly, *ACE2* deletion in C. albicans results in the upregulation of several *ERG* genes involved in sterol biosynthesis (42), and thus, we reasoned that *ERG6* could be upregulated in resistant mutant R3.3, thus resulting in tomatidine resistance. *ERG6* expression was, however, not significantly altered in R3.3 compared to that in the parent, and thus, the basis of resistance by *ACE2* remains unresolved.

In comparison to previous reports from studies with S. cerevisiae that suggest that Erg6 is a tomatidine target (19), our forward genetic approach evidenced a direct interaction between Erg6 and tomatidine, with a single substitution of well-conserved amino acids (G132D or D249G) being sufficient to confer resistance. As a loss of function in Erg6 results in increased susceptibility to tomatidine, the two mutations did not drastically affect Erg6 function (ergosterol was detected, and amphotericin B susceptibility remained unaffected). In the absence of an X-ray structure for C-24 sterol methyltransferase (Erg6), a previous functional analysis using bioinformatics analysis, mechanism-based inactivation, and site-directed mutagenesis experiments identified functionally important residues in three regions (Fig. 5B) (30). The two residues (G132D and D249G) were not included in these analyses, and their effect on the kinetics of the C-methylation reaction remains to be determined. To address the mechanism of Erg6 inhibition by tomatidine, studies of a known Erg6 inhibitor, 25-azalanosterol, have suggested noncompetitive binding to a site different from the sterol binding site in the active center which leads to conformational changes deleterious to the catalytic reaction (30, 43). The two identified residues could mediate direct interactions with tomatidine, and nonconserved changes in these positions may result in a decreased affinity to the drug.

An important issue concerning the potential of tomatidine as an antifungal drug is its resilience to efflux pump-mediated mechanisms, which are commonly acquired by azole exposure. We showed here that tomatidine is the substrate of Cdr1 in C. albicans. By extrapolation, we can hypothesize that the absence of activity against C. glabrata, which is known to possess intrinsic resistance to antifungals, is related to its potent efflux machinery. Nevertheless, we showed, using matched azole-sensitive and azole-resistant clinical isolates, that the common mechanism of resistance to azole through *TAC1* hyperactivity had a limited effect on tomatidine susceptibility. This reduced cross-resistance and the increased fluconazole susceptibility of a tomatidine-resistant strain (via G132D) raised the question about the potential therapeutic advantage of the combined use of the two drugs. Our analysis revealed an additive effect of the combination against a wild-type strain of C. albicans. In a recent study (44), a screen for molecules with synergistic activity when they are used in combination with fluconazole against a C. albicans azole-resistant strain (with *ERG11* and *TAC1* mutations) identified a synergistic interaction with tomatidine, thus confirming the strong potential for the use of tomatidine in drug combination therapies.

### *In vivo* activity of tomatidine.

Our initial choice to screen a library of NPs was guided by the intrinsic properties of these natural compounds compared to those of synthetic products, namely, their immense chemical diversity, target specificity, and intrinsic ability to permeate cells. The critical step in the development of new antifungal agents is to validate promising *in vitro* characteristics *in vivo*, and by choosing NPs, we were hoping to bypass this bottleneck. *In vivo* studies have validated that the target of tomatidine (Erg6) is required for virulence in both mice and insect minihost models of C. albicans systemic infection (45; data not shown). Tomatidine is a highly hydrophobic sterol-like molecule difficult to dissolve using common aqueous solvents. First attempts to demonstrate the *in vivo* activity of tomatidine in an animal model of C. albicans systemic infection using (co)solvents (dimethyl sulfoxide [DMSO], ethanol) or cyclodextrin complexation (hydroxypropyl beta-cyclodextrin) or surfactant (polyoxyl 35 castor oil) were not successful. We next reasoned that other types of drug formulations could be more successful. Several studies have used nanoparticle preparations to increase drug efficacy *in vivo*. For example, Moazeni et al. (46) have reverted the *in vitro* azole resistance of Candida spp. with solid lipid nanoparticles prepared with fluconazole. Inspired by this work, we prepared a nanoparticle-based tomatidine formulation. This formulation, which did not affect the *in vitro* activity of tomatidine and did not modify its chemical structure, was injected i.p. and significantly decreased the fungal burden in the kidney compared to that achieved with placebo. Further studies are necessary to understand the bioavailability and biodistribution of tomatidine in mice; however, our work clearly raises the possibility that tomatidine is a novel potential therapeutic antifungal agent.

## MATERIALS AND METHODS

### Yeast strains, culture, and growth media.

The yeast and bacterial strains used in the study are listed in Table S1 in the supplemental material. NPs were dissolved in DMSO to 10 μg/ml. Tomatidine (tomatidine-HCl) was purchased from Phytolab (GmbH), dissolved in DMSO at a 2 mM concentration, heated for 20 min at 50°C for solubilization, and stored at −20°C until usage.

### Antimicrobial susceptibility testing. (i) Antifungal broth microdilution assays.

Antifungal susceptibility testing was carried out on the basis of EUCAST protocols with slight modifications (47). Briefly, yeast strains were cultivated overnight at 30°C under constant agitation in 1% yeast extract, 2% peptone, and 2% glucose (YEPD) medium. Cultures were diluted to a density of 2 × 10^5^ cells per ml in RPMI medium (catalog number R8755; Sigma-Aldrich) or yeast nitrogen base (YNB; MPbio, Santa Ana, CA, USA) that was buffered to an adequate pH with HCl or NaOH and that had a final glucose concentration of 2%. Compounds from the NP library were dissolved to a final concentration of 10 mg/ml in DMSO. The final DMSO concentration was 1%. Twofold serial dilutions with concentrations ranging from 32 to 1 μg/ml were prepared. The plates were incubated at 35°C for 24 h, and then the MICs were read with a spectrophotometer plate reader set at 450 nm. The MIC was defined as the drug concentration at which the optical density (OD) was equal to or decreased by more than 50% from that of the drug-free culture. For S. cerevisiae MICs, the medium (YNB) was complemented with complete supplement mixture (CSM; Mpbio, Santa Ana, CA, USA) according to the supplier's instructions. Assays were performed in triplicate.

For the detection of an effect of the combination of tomatidine and fluconazole, a checkerboard assay was set up in YNB (pH 7) with 2-fold dilutions of fluconazole (0.008 to 0.5 μg/ml, 1 to 64 μg/ml) and tomatidine (0.08 to 40 μM, 0.02 to 10 μM). The use of combinations of the drugs at different concentrations enables determination of a fractional inhibitory concentration (FIC) index, as described in reference 48. Drug combinations that give rise to a growth reduction of more than 50% are then identified, and the associated FIC index determines the properties of the combination. A FIC index of between 2 and 0.5 indicates an additive effect, while a FIC index of <0.5 indicates a synergetic effect. Average values were calculated from three replicates.

### (ii) Antifungal serial dilution susceptibility assay.

Drug susceptibility testing was also performed on solid YEPD agar plates containing specific drug concentrations or vehicle (1% DMSO). Tenfold serial dilutions of cells were spotted starting with a cell concentration of 10^6^ cells/ml. Assays were performed at least in duplicate.

### (iii) Antifungal biofilm susceptibility assay.

Antifungal susceptibility tests were conducted on C. albicans biofilms according to a published protocol (49) with 48 h of biofilm formation and 48 h of antifungal treatment. Briefly, an aliquot of a 100-μl cell suspension (10^6^ cells/ml) per well prepared in RPMI medium with 0.2% glucose (pH 7) was deposited in each well of a 96-well plate, and the plate was incubated at 37°C for 48 h to allow biofilm formation. The wells were then washed twice with phosphate-buffered saline (PBS). Twofold serial dilutions of the compounds with concentrations ranging from 50 to 1.56 μg/ml were prepared, and the compounds were added to the wells containing the biofilms. The plates were incubated again for 48 h at 37°C and then washed twice with PBS. Measurement of the metabolic activity of the sessile cells was performed using a colorimetric assay with 2H-tetrazolium,2,3-bis(2-methoxy-4-nitro-5-sulfophenyl)-5-[(phenylamino)carbonyl]-hydroxide salt (XTT; catalog number X4626; Sigma-Aldrich). The plates were read with a spectrophotometer plate reader at 492 nm. The MIC was defined as the drug concentration at which the optical density value was equal to or less than 50% of the one for the drug-free biofilm. Assays were performed in duplicate.

### (iv) Antifungal drug time-kill assay.

The time-kill assay was performed as follows. Cells were cultured overnight in YEPD at 30°C, adjusted to 2 × 10^5^ cells/ml in YEPD, and submitted to the corresponding concentration of the drugs, their combination, or the solvent. After 0, 4, 8, and 24 h of incubation with the drug at 30°C under agitation in a 3-ml liquid volume, cell viability was determined by plating the cells on a YEPD agar plate for 16 h at 34°C and counting the number of CFU of the colonies. The drug was determined to have a fungicidal effect when at least a 2-fold log decrease in the number of CFU per milliliter from the initial cell density was measured.

### (v) Antibacterial susceptibility assay.

Antibacterial susceptibility testing was carried out on the basis of CLSI approved standard M7-A7 using the microdilution method with cation-adjusted Mueller-Hinton (CAMH) broth. Briefly, overnight Escherichia coli (ATCC 25922) cell cultures were adjusted to a McFarland 0.5 standard (10^8^ cells/ml) with NaCl. The final cell concentration was 3 × 10^5^ CFU/ml. Twofold serial dilutions of the drugs at concentrations ranging from 64 μg/ml to 2 μg/ml were prepared. The microplates were incubated at 37°C for 24 h, and then the MICs were read with a spectrophotometer plate reader at 450 nm. The MIC was defined as the drug concentration at which the optical density was equal to or decreased more than 50% from that of the drug-free culture. Assays were performed in duplicate. Drugs were tested at the pH at which antifungal activity was detected during the microdilution susceptibility screen (pH 7 was used if the drug was active at both pH values).

### Cell cytotoxicity assay.

A cell cytotoxicity assay were performed according to a standard procedure with sulforhodamine B (SRB) as the reporter (50). HeLa cells (CCL-2; ATCC, Manassas, VA, USA) were cultured in Dulbecco modified Eagle medium with 10% fetal bovine serum at 37°C with 5% CO_2_. Ninety-six-well plates were filled with a seeding density of 10^4^ cells/well. After 24 h of growth (day 1), the cells were washed twice with PBS, 2-fold serial dilutions (starting at 100 μg/ml) of the compounds were added to the cells, and the cells were incubated for 48 h. The starting amount of cells was monitored by fixing the cells on day 1. On day 3, all cells were washed twice with PBS and then fixed and labeled as described above for the standard procedure. The optical density at 492 nm (OD_492_) was measured, and the percentage of cells killed could be determined using the following formula: 100 − (OD on day 3/OD on day 1) × 100. The 50% lethal dose (LD_50_) corresponded to the concentration at which at least 50% of the cells were killed. Assays were performed in duplicate. The selectivity index (SI) was then calculated by dividing the LD_50_ by the MIC against C. albicans.

### Hierarchical clustering of activity profiles.

The MICs of the 40 NPs active against the 7 yeast strains were used to generate the heatmap, and the pH of the activity was indicated by use of a color scheme. NPs that showed activity at both pHs were labeled as being active at neutral pH. Cluster analyses were performed by calculating the distance matrix using the Euclidean method, followed by Ward (Ward.D) hierarchal clustering using the gplots package in R (version 3.3.2).

### Calcofluor white staining.

Yeast cells were grown overnight and washed twice with PBS. Cells (10^5^) were resuspended in 200 μl RPMI medium with 0.2% glucose in a 96-well plate and incubated for 3 h at 37°C. Ten microliters of calcofluor white stain (Sigma-Aldrich) was added to the well. After 10 min at room temperature, 4 μl of the cell suspension was mixed with 2 μl of Mowiol mounting medium (Sigma-Aldrich), and directly thereafter, fluorescence microscopy was performed with a Zeiss Axioplan 2 microscope (Zeiss, Oberkochen, Germany).

### Sterol content analysis. (i) Total sterol extraction.

About 2 × 10^5^ cells/ml were cultured for 16 h at 30°C under agitation in 15 ml YEPD supplemented with tomatidine at the concentrations indicated above (and with DMSO at a final concentration of 1%). The cells were treated with trichloroacetic acid (TCA) to a final concentration of 5% to stop metabolism, and the cells were incubated for 10 min on ice. Harvested cells were then washed twice: first in 5% TCA in distilled water and then in distilled water to remove the traces of the YEPD medium. The cells were resuspended in 3 ml of distilled water, and 10^9^ cells were used to perform total sterol extraction, as described in reference 51, and to determine the amounts of total cellular sterols (esterified and nonesterified). The cells were resuspended in 1 ml 60% KOH in a screw-cap glass tube, to which 1 ml of 0.5% pyrogallol-containing methanol and 1 ml of methanol were added. The tubes were heated at 85°C for 2 h and returned to room temperature. The sterols were extracted three times with 2 ml of petroleum ether (high boiling point). The combined petroleum ether phases were dried under an N_2_ flow, resuspended in 1:1 (vol/vol) methanol-chloroform, and sonicated for 5 min for further analysis by gas-liquid chromatography–mass spectrometry (GLC-MS).

### (ii) GLC-MS analysis.

Sterols were analyzed by GLC-MS as described in reference 51. *ERG4* and *ERG6* deletion mutant extracts were used to determine the positions of the known sterols.

### Tomatidine formulation.

A nanosuspension was obtained by adding 6 mg of tomatidine, 300 μl 2% (wt/vol) d-α-tocopherol polyethylene glycol 1000 succinate (TPGS; Sigma-Aldrich) as a stabilizer, and 700 μl of purified water as a nonsolvent in a 2-ml tube (tomatidine-TPGS [50:50]). Wet milling with 579 mg of zirconium beads (tubes prefilled with 2.0 ml 0.5-mm zirconium beads, triple pure, high impact; BeadBug; Sigma-Aldrich) was performed for 70 h on a Genie 2 vortex apparatus. Then, the nanosuspensions were frozen by dipping the tubes in liquid nitrogen. Subsequently, the nanosuspensions were lyophilized for 48 h using a Christ Alpha 2-4 LD Plus freeze-dryer. The particle size distribution was determined by dynamic light scattering (DLS) using a Zetasizer 3000HSA particle analyzer. The samples were dispersed in a 9/10 volume of filtered purified water and stirred for 20 min with a vortex mixer to ensure a uniform dispersion free of aggregates. A 1/10 volume of 10× PBS was added prior to injection.

### Transmission electron microscopy (TEM).

C. albicans strain CAF2-1 was grown in YNB liquid cultures for 2 h at 37°C (in 15-ml plastic tubes). Next, miconazole (1 mg/ml in DMSO) was added at a concentration of 10 μg/ml, and the cultures were grown for 18 h to evaluate the cytotoxic effect of this commercial product on the yeast strain. The cytotoxic effect of tomatidine was evaluated by use of the same experiment, except that this compound (1 mg/ml in DMSO) was added at a concentration of 20 μM and the cultures were grown for 18 h. Cells were prepared as described in reference 12. Thin sections were observed with a transmission electron microscope (Philips CM10) with a Mega View II camera. Control cells were obtained in the same way but did not receive drug treatment.

### Selection of NPs.

A smart chemical library containing 199 natural products (NPs) with potential antifungal activity was constructed. Among these compounds, 53% were previously isolated from crude plant extracts that presented antifungal activity. The bioguided process of isolation of these compounds was performed by bioautography using wild-type and genetically modified strains of C. albicans (12). In parallel, NPs with (i) structures closely related to those of NPs possessing antifungal activity from published sources and (ii) unknown antifungal activity were selected from commercial catalogues and acquired (the compounds are listed in Table S1 in the supplemental material). The identity and the purity of the commercial compounds obtained were systematically determined by nuclear magnetic resonance (NMR) and high-resolution mass spectrometry (HRMS) analysis.

### Genome-wide transcriptional analysis. (i) RNA extraction and processing.

An overnight YEPD culture of the C. albicans SC5314 strain was diluted 1:200 in 5 ml YEPD medium and incubated under agitation at 30°C until early exponential growth phase (OD_540_ = 0.3). Fifty microliters of solvent (DMSO) or 250 μM tomatidine (diluted in DMSO) was added to the culture to reach a concentration of 1% DMSO and 2.5 μM tomatidine. Total RNA was extracted after 1 or 3 h tomatidine-solvent exposure by mechanical disruption of the cells with glass beads as previously described (52). Experiments were carried out in triplicate with 12 samples. Total RNA extracts were treated with DNase using a DNA-free kit (Ambion-Life Technologies, Zug, Switzerland), and RNA quality and integrity were verified with a fragment analyzer automated CE system (Advanced Analytical). One microgram of RNA was used to create sequencing libraries through a standard Illumina TruSeq stranded mRNA protocol. Each library (sample) received a different index, enabling several libraries to be multiplexed. Before RNA sequencing, the libraries were analyzed with a fragment analyzer to assess their quality and fragment size and with a Qubit fluorometer (Invitrogen) to determine the cDNA concentration. Libraries were kept at −20°C until sequencing.

### (ii) RNA sequencing.

The 12 libraries were run on an Illumina HiSeq platform (HiSeq2500). Sequencing data were processed using Illumina Pipeline software. Reads were filtered and trimmed, and the counts were aligned to the C. albicans SC5314 reference genome using the CLC workbench pipeline. The number of read counts per gene locus was extracted.

### (iii) RNA-seq data analysis.

Data normalization and gene expression analysis were performed in R (version 3.2.3), using Bioconductor packages (as described in reference 24). The read count data were normalized using the TMM (trimmed mean of M values) method available in the R package edgeR (53) and transformed into log_2_ counts per million by the Voom method from the R package Limma (54). This package was then used to apply a linear model with one factor per condition (4 conditions, consisting of untreated for 1 h, tomatidine treated for 1 h, untreated for 3 h, and tomatidine treated for 3 h [all in triplicate]) to the transformed data. Two contrasts representing the difference between tomatidine-treated and untreated cells at each drug exposure time (1 and 3 h) were extracted from the linear model to result in a moderated *t* statistic for all genes expressed.

### qPCR analysis.

Total RNA (the same RNA samples used for the RNA-seq experiments; 12 samples representing 4 conditions in 3 biological replicates) were treated with DNase, and 1 μg of treated RNA was used as the template for cDNA synthesis using a high-fidelity cDNA synthesis kit (Roche Diagnostics, Switzerland). To determine relative gene expression, real-time quantitative PCR (qPCR) was performed using primers, TaqMan probes (modified with 6-carboxyfluorescein and 6-carboxytetramethylrhodamine), and iTaq supermix with carboxy-X-rhodamine (Bio-Rad AG, Switzerland) in a StepOnePlus real-time PCR system (Applied Biosystems-Life Technologies, Switzerland). Each reaction was run in duplicate. The primers and probes are listed in Table S3. Relative transcript quantities were assessed using the 2^−ΔΔ*CT*^ threshold cycle (*C_T_*) method (55) to determine the level of expression, which was normalized to that of *ACT1* as a reference gene.

### C. albicans *ERG6* deletion strain construction.

To delete the first allele of *ERG6*, two fragments of 571 bp and 569 bp of the flanking 5′ and 3′ untranslated regions, respectively, were PCR amplified from SC5314 DNA with the following primer pairs: primer pair ERG6_5For_KPN1 and ERG6_5Rev_Xho1 and primer pair ERG6_3For_SacII and ERG6_3Rev_SacI. These primers contain restriction sites in order to insert the two amplicons sequentially in pSFS2A (56). The plasmid obtained (pSD1) was then digested with ScaI and transformed into C. albicans SC5314. The yeasts were transformed by a lithium acetate procedure described previously (57).

Transformants were positively selected on a YEPD plate containing 200 μg/ml nourseothricin (Nour; Werner Bioagents, Germany). The Nour selective cassette was then removed by growing cells in YEPD medium containing 2% maltose. Nour-susceptible cells were used to delete the second allele. The same strategy was repeated for the second allele, but in order to achieve it, a different 3′ end region homologous to *ERG6* (at the end of the coding DNA sequence [CDS] and upstream of the first allele, 343 bp) was amplified with primers ERG6_3CDSFor_SacII and ERG6_3CDSRev_SacI. The resulting construct was named pSD4.

Nour-resistant transformants were phenotypically screened using a simplified ergosterol extraction and detection method (58). An alteration of the expected UV spectrophotometric sterol profiles was detected in some transformants, with an additional peak of absorbance at 230 nm indicating a perturbation in the ergosterol pathway and thus suggesting the deletion of the second *ERG6* allele. GLC-MS analysis confirmed the loss of *ERG6* function, with zymosterol being the most abundant sterol (data not shown).

### S. cerevisiae forward genetic screen. (i) Deletion of *MSH2* in S. cerevisiae DSY4743.

The *MSH2 PDR5* deletion strain was constructed from a *PDR5* deletion strain (DSY4743) using a PCR-based gene deletion approach described previously (61). Primers For_msh2_Sc and Rev_msh2_Sc were used to amplify the *HIS3* selection marker. The *HIS3*-containing amplicons were purified using a NucleoSpin gel and PCR cleanup kit (Macherey-Nagel, Düren, Germany) according to the manufacturer's instructions and used to transform S. cerevisiae *pdr5*Δ (DSY4743) using the standard lithium acetate protocol.

### (ii) Selection of resistant mutants.

Overnight cultures of cells of the *msh*2Δ *prd5*Δ strain (strain P1) were plated on solid medium containing one of several tomatidine concentrations and incubated for 2 to 7 days. One pop-out mutant (mutant R1) was identified, and resistance to tomatidine was confirmed using the MIC broth dilution method. The other tomatidine-resistant strains were identified as described previously by Ojini and Gammie (29) with few modifications. The *msh*2Δ *prd5*Δ strain (P1) was grown to saturation in 5 ml of YEPD medium at 30°C. Overnight cultures were diluted 1:200 in YEPD medium containing 50 μg/ml of ampicillin and grown in the presence of 10 μM tomatidine in 96-well microtiter plates (Costar) in a shaking incubator at 30°C for 72 h. The cells were diluted 1:200 in the respective medium, distributed into new 96-well plates, and grown for 48 h at 30°C in the absence of the drug. The cultures were then diluted 1:200 in medium containing tomatidine in a new 96-well plate, and the optical density at 540 nm was recorded at six different time points (0, 16, 20, 24, 38, and 42 h) over a 42-h period. The isolates in several wells were selected on the basis of their growth profiles with a high optical density in the presence of drug after 40 h, plated onto YEPD containing 7.5 μM tomatidine, and incubated for 4 days at 30°C. Resistant pop-outs were cultured overnight, and resistance was retested by the broth dilution method. Four resistant strains arising from 2 different cultures (strain R2 and strains R3.1, R3.2, and R3.3) were identified and, together with the R1 mutant, submitted to whole-genome sequencing.

### (iii) Whole-genome sequencing.

Cells of the five tomatidine-resistant mutants and the parental strain were grown overnight in YEPD medium at 30°C under constant agitation. Genomic DNA was extracted from the yeast using a Gentra Puregene yeast/bacteria kit (Qiagen, Hilden, Germany) with RNase treatment. The DNA concentration was verified by use of a Qubit (version 2.0) fluorometer (Thermo Fisher) and adjusted to 10 ng/ml for whole-genome sequencing. DNA quality was verified with a fragment analyzer (Advanced Analytical Technologies, Ankeny, IA, USA). Whole-genome sequencing was performed at Fasteris SA (Plan-les-Ouates, Switzerland) using a TrueSeq Nano DNA library preparation and an Illumina MiSeq system. Paired-end reads of 250 bp were performed, giving an average coverage of each genome of 118 times. The sequencing data were analyzed using the CLC Genomics Workbench (version 9.5.2) program (Qiagen). The sequence reads were mapped to the S288C reference genome. The average percentage of mapped reads was 93%. Mutations were identified using the variant detector option by comparing the sequences of the genomes of the P1 and tomatidine-resistant strains to the sequence of the reference genome. The functional consequences option was used to identify amino acid changes. To identify mutations specific to the resistant strains, the sequences of the variants of the tomatidine-resistant strains were compared to the sequence of the P1 strain using the compare variants option. Only nonsynonymous mutations were taken into account, with a frequency of ≥50% of reads for insertions and deletions (indels) and a frequency of ≥90% of reads for single nucleotide variants (SNV) being used.

### (iv) Construction of S. cerevisiae *ERG6* point mutant strains.

To introduce specific point mutations into *ERG6* in S. cerevisiae, a clustered regularly interspaced short palindromic repeat (CRISPR)-Cas9 genome editing system was used as described previously (31). All primers are listed in Table S3. A 20-nucleotide guide sequence was selected using an online tool named CHOPCHOP (http://chopchop.cbu.uib.no/). PAM 11 (position 251849) and PAM 19 (position 252596) were chosen for the D249G mutation (position 252245) and G132D mutation (position 252596), respectively. The two repair fragments were constructed as described previously (59). All fragments were purified using a NucleoSpin gel and PCR cleanup kit (Macherey-Nagel).

Genome editing was performed by cotransformation of the pMEL10 guide and the repair fragments into S. cerevisiae IMX581, and selection was carried out in YNB agar lacking uracil. Verification that the mutations were introduced was performed by PCR amplification of *ERG6* (with primers Erg6_verif_for and Erg6_verif_rev; Table S3) and by sequence analysis as described above. The guide RNA plasmid pMEL10 were removed by counterselection pressure with 5-fluoroorotic acid (5-FOA; Toronto Research Chemicals [TRC]), and the effect of the mutations on tomatidine susceptibility was verified by the broth dilution method.

### Mouse experiments and ethics statement.

All animal experiments were performed at the University Hospital Center of Lausanne, Lausanne, Switzerland, with approval through the Institutional Animal Use Committee, Affaires Vétérinaires du Canton de Vaud, Switzerland (authorization no. 1734.3), according to decree 18 of the federal law on animal protection. Female BALB/c mice (age, 8 weeks; Charles River France) were housed in ventilated cages with free access to food and water. The SC5314 strain was grown overnight under agitation at 30°C in YEPD medium, subsequently diluted 100-fold in YEPD medium, and grown again overnight under agitation at 30°C. Overnight cultures were washed twice with PBS and resuspended in 5 ml PBS. The concentration of each culture was measured by determination of the optical density, and each strain was diluted in PBS to the desired concentration (4 × 10^5^ CFU/ml). Mice were injected through the lateral tail vein with 250 μl of a cell suspension containing 1.6 × 10^6^ cells/ml. At 7, 24, and 31 h postinfection, a tomatidine formulation or placebo was administered by intraperitoneal (i.p.) injection in a volume of 200 μl. At 48 h postinfection, the kidneys were recovered and weighed, and the number of CFU was determined as previously described (60). The number of CFU per gram of kidney was determined. Outlier analysis was first performed in GraphPad Prism software using default parameters (robust regression and outlier removal [ROUT] with a coefficient Q = 1%), and the final number of individuals per group was 10 for placebo-treated mice and 9 for tomatidine-treated mice. Statistical analyses of the differences between the numbers of CFU were performed using the Mann-Whitney test. The weight and temperature of the animals were monitored daily.

### Accession number(s).

All reads were deposited in the Gene Expression Omnibus (GEO) database under accession number GSE96965. Genome data have been deposited at NCBI under BioProject accession number PRJNA380059.

## Supplementary Material

Supplemental material
