# Supplementary material for: Identification and Mode of Action of a Plant Natural Product Targeting Human Fungal Pathogens
Source: Antimicrob Agents Chemother. 2017 Aug 24;61(9):e00829-17. doi: 10.1128/AAC.00829-17 (PMC5571344; doi:10.1128/AAC.00829-17)
Supplement: Supplemental material [file supp_61_9_e00829-17__index.html]

Supplemental material 

# Identification and Mode of Action of a Plant Natural Product Targeting Human Fungal Pathogens

## Supplemental material

- Supplemental file 1 -

  Supplemental Tables S1 to S4 and Figures S1 to S7

  PDF, 5.2M
- Supplemental file 2 -

  Data Set S1: tomatidine RNAseq data analysis.

  XLSX, 319K
- Supplemental file 3 -

  Data Set S2: GSEA analysis with tomatidine gene set and gene lists used for GSEA.

  XLSX, 125K
- Supplemental file 4 -

  Data Set S3: lists of nonsynonymous mutations obtained per resistant strain in the forward genetic screen in *S. cerevisiae*.

  XLSX, 25K
