# Supplementary material for: Identification and Mode of Action of a Plant Natural Product Targeting Human Fungal Pathogens
Source: Antimicrob Agents Chemother. 2017 Aug 24;61(9):e00829-17. doi: 10.1128/AAC.00829-17 (PMC5571344; doi:10.1128/AAC.00829-17)
Supplement: Supplemental material [file AAC.00829-17_zac009176507s1.pdf]

Supplementary Tables

Table S1: List of the 209 NPs that were screened for antifungal activities against *C. albicans.*

Name	Fraction	Source	CAS number	Type of molecule
2-Anthracenecarboxylic acid	F79B	*Morinda tomentosa*	117-78-2	anthraquinone
morindone	F102-103	*Morinda tomentosa*	478-29-5	anthraquinone
2-hydroxy-3-methyl- anthraquinone	F85	*Morinda tomentosa*	17241-40-6	anthraquinone
iridoid glycoside scandoside	F7	*Morinda tomentosa*	-	anthraquinone
lucidin primeveroside	F52	*Morinda tomentosa*	9706-59-0	anthraquinone
rubiadin primveroside	F53	*Morinda tomentosa*	26388-47-6	anthraquinone
damnacanthole	F61A	*Morinda tomentosa*	477-83-8	anthraquinone
2-hydroxymethyl-1-methoxyl-anthraquinone	F61B	*Morinda tomentosa*	-	anthraquinone
alizarin-1-methyl ether	F68-69	*Morinda tomentosa*	6170 06 5	anthraquinone
morindone 3-O-β-primeveroside	F71-73	*Morinda tomentosa*	60450-21-7	anthraquinone
rubiadine-1-methyl ether	F79A	*Morinda tomentosa*	7460-43-7	anthraquinone
lucidine ω-methyl ether	F90-91	*Morinda tomentosa*	-	anthraquinone
tectoquinone	F99A	*Morinda tomentosa*	84-54-8	anthraquinone
soranjidiol	F99b	*Morinda tomentosa*	518-73-0	anthraquinone
morindoquinone	F105	*Morinda tomentosa*	-	anthraquinone
hydroxy-2-methylanthraquinone	F107	*Morinda tomentosa*	6268 09 3	anthraquinone
avocadyne	F16	*Persea americana*	24607-05-4	fatty alcohol
avocadène	F19	*Persea americana*	24607-08-7	fatty alcohol
avocadyne acetate	-	*Persea americana*	-	fatty alcohol
avocadene acetate	-	*Persea americana*	-	fatty alcohol
alpha-hederin	-	*Schefflera systila*	27013-91-8	saponin
plumbagin	-	Sigma	481-42-5	naphtoquinone
alpha-hederin	-	Sigma	27013-91-8	saponin
Combretastatin A4	-	Sigma	117048-59-6	stilbene

Name	Fraction	Source	CAS number	Type of molecule
arjunolic acid	-	*Conostegia speciosa*	465-00-9	triterpene
Tovopyrifolin C	N°33	*Monnina obtusifolia*	34211-53-5	xanthone
Glc-3medicagenic acid	N°58	*Dolichos kilimandscharicus*	-	saponin
9*H*-Xanthen-9-one, 1,4,5-trihydroxy-3-(3-methyl-2-buten-1-yl)-	N°117	*Garcinia livingstonei*	136364-72-2	xanthone
9*H*-Xanthen-9-one, 1,7-dihydroxy-4-methoxy-	N°122	*Polygala nyikensis*	87339-76-2	
Phenol, 4-(3,4-dihydro-7-methoxy-2*H*-1-benzopyran-2-yl)-2-methoxy-	N°155	*Mariscus psilostachys*	116498-59-0	
2-Propen-1-one, 1-(2,4-dihydroxy-6-methoxy-3,5-dimethylphenyl)-3-phenyl-	N°190	*Myrica serrata*	65349-31-7	
monotesone A	N°338	*Monotes engleri*	208596-55-8	flavanone
Prenyletin methyl ether	N°343	*Vepris dicarpella*	13544-37-1	coumarin
O-Methyllawsone	N°345	*Swaertia calycina*	2348-82-5	naphtoquinone
thymol	N°551	*Mosla chinensis*	89-83-8	monoterpene
negletein	N°552	*Mosla chinensis*	29550-13-8	flavanone
1-Naphthalenecarboxylic acid, 5-[2-(3-furanyl)-2-oxoethyl]decahydro-1,4a-dimethyl-6-methylene-, methyl ester, (1*R*,4a*S*,5*R*,8a*S*)-	N°629	*Potamogeton pectinatus*	329371-63-3	
cuspidiol	N°697	*Fagara zanthoxyloides*	51593-96-5	monomeric phenyl propanoid
8-Acetonyldihydronitidine	N°701	*Fagara zanthoxyloides*	80330-39-8	
Dihydrochelerythrine	N°705	*Fagara zanthoxyloides*	6880-91-7	alkaloids
Swartziarboreol B	F-58	*Swartzia simplex*	173357-22-7	cassane diterpene
simplexene B	F-72	*Swartzia simplex*	New	cassane diterpene
simplexene D	82.A	*Swartzia simplex*	New	cassane diterpene
11,12-dihydroxy-15,16-dihydroswartziarboreol C	ss F-83	*Swartzia simplex*	1016630-54-8	cassane diterpene
Swartziarboreol E	ss F-35	*Swartzia simplex*	173357-25-0	cassane diterpene
Swartziarboreol A	ss F-51	*Swartzia simplex*	173357-21-6	cassane diterpene

Name	Fraction	Source	CAS number	Type of molecule
11,12-dihydroxy-8,11,13,15-cassatetraen-17,16-olide	ss F-94.G	*Swartzia simplex*	-	cassane diterpene
dehydroabietic acid (15B)	PD-139	*Plagiochila delavayi*	1740-19-8	diterpene
diplophyllolide A	F15(b) Poly	*Chiloscyphus polyanthus*	56632-84-9	diterpene
waltherione G	F66-1	*Waltheria Indica*	-	quinoline alkaloids
waltherione F	F76	*Waltheria Indica*	-	quinoline alkaloids
8-deoxoantidesmone	F49	*Waltheria Indica*	-	quinoline alkaloids
waltherione E	F61	*Waltheria Indica*	-	quinoline alkaloids
pterostilbene	-	commercial	537-42-8	stilbene
hederagenin commercial	-	extrasynthèse	465-99-6	triterpene
simplexene A	67.B3	*Swartzia simplex*	-	cassane diterpene
(5*S*,10*S*)-11,15(*S*)-dihydroxy-12-methoxyswartziarboreol G	67.B4	*Swartzia simplex*	1830306-56-3	cassane diterpene
15,16-Dihydroswartziarboreol C	58.C	*Swartzia simplex*	864179-56-6	cassane diterpene
saponin FLO A	-	*Swartzia simplex* leaves		saponin
Lapachol	-	commercial	84-79-7	naphtoquinone
Pulsatilla saponin D	Fr28	*Odondatenia puncticulosa*	68027-15-6	saponin
3β-O-[β-D-xylopyranosyl-(1→3)]-α-L-rhamnopyranosyl-(1→2)-[β-D- glucopyranosyl-(1→4)]-α-L-arabinopyranosyl]hederagenin	Fr30	*Odondatenia puncticulosa*	-	saponin
Afrormosine	-	*Swartzia simplex*	550-79-8	Flavonoid
simplexene C	-	*Swartzia simplex*	New	cassane diterpene
Swartziarboreol C	-	*Swartzia simplex*	173357-23-8	cassane diterpene
simplexene E	-	*Swartzia simplex*	New	cassane diterpene
Lucidin	-	Phytolab	21087-77-4	anthraquinone
Urushiol (15:3)	-	Phytolab	478-08-0	phenol
Rubiadin -	-	Phytolab	83543-37-7	anthraquinone

Name	Fraction	Source	CAS number	Type of molecule
Anisodamine hydrobromide	-	Phytolab	55449-49-5	alkaloids
Chrysophanol	-	Phytolab	481-74-3	anthraquinone
Dictamnine	-	Phytolab	484-29-7	furoquinoline alkaloid
Garcinone C	-	Phytolab	76996-27-5	xanthone
Indirubin	-	Phytolab	479-41-4	Indol alkaloid
6-Gingerol	-	Phytolab	23513-14-6	aryl-alcanones
quercetrin 7-O-gucoside	-	Phytolab	491-50-9	flavonol
Muscimol	-	Phytolab	2763-96-4	isoxazole
Neoandrographolide	-	Phytolab	27215-14-1	diterpene
Anisodine hydrobromide	-	Phytolab	76822-34-9	tropanic alkaloid
Cimifugin	-	Phytolab	37921-38-3	Chromone
Dihydrocapsaicin	-	Phytolab	19408-84-5	Amide
Garcinone D	-	Phytolab	107390-08-9	xanthone
dihydrotanshinone I	-	Phytolab	87205-99-0	diterpene
8-Gingerol	-	Phytolab	23513-08-8	aryl-alcanones
Rubiadin	-	Phytolab	117-02-2	anthraquinone
Rubiadin 1-methyl ether	-	Phytolab	7460-43-7	anthraquinone
Obacunone	-	Phytolab	751-03-1	limonoide
Arecoline hydrobromide	-	Phytolab	300-08-3	pyridimique alcaloid
Coptisin chloride	-	Phytolab	6020-18-4	benzylisoquinoline alkaloid
Dihydroberberine	-	Phytolab	483-15-8	benzylisoquinoline alkaloid
Corynoline	-	Phytolab	18797-79-0	benzylisoquinoline alkaloid
Cryptotanshinone	-	Phytolab	35825-57-1	diterpene
Swertiamarine	-	Phytolab	17388-39-5	secoiridoid
Ruscogenin	-	Phytolab	472-11-7	triterpene
	-	Phytolab	15619-74-9	
Aristolactam	-	Phytolab	13395-02-3	benzylisoquinoline alkaloid

Name	Fraction	Source	CAS number	Type of molecule
Cordycepin	-	Phytolab	73-03-0	nucleoside derivative
Californidine perchlorate	-	Phytolab	17939-31-0	tropanic alkaloid
Costunolide	-	Phytolab	553-21-9	sesquiterpene lactone
Capsaicin	-	Phytolab	404-86-4	alphatic amide
Ginkgolic acid C13:0	-	Phytolab	20261-38-5	annacardic acid derivative
Tanshinone I	-	Phytolab	568-73-0	diterpene
Rutaecarpine	-	Phytolab	84-26-4	
Palmatine chloride	-	Phytolab	10605-02-4	benzylisoquinoline alkaloid
(+)-Bicuculline	-	Phytolab	485-49-4	phthalide-isoquinoline alkaloid
Ajmalicine	-	Phytolab	483-04-5	benzylisoquinoline alkaloid
Canadine	-	Phytolab	522-97-4	benzylisoquinoline alkaloid
Aloe emodin	-	Phytolab	481-72-1	anthraquinone
Dodeca 2E,4E,8Z,10E,Z- tetraenoic acid isobutylamide	-	Phytolab	866602-52-0	isobutylamide
Ginkgolic acid C15:1	-	Phytolab	22910-60-7	annacardic acid derivative
Tanshinone IIA	-	Phytolab	568-72-9	diterpene
Sarsasapogenin	-	Phytolab	126-19-2	saponin
Pennogenin tetraglycoside	-	Phytolab	68124-04-9	saponin
Trigonelline chloride	-	Phytolab	6138-41-6	pyridine alkaloid
L-(±)-Alliin	-	Phytolab	17795-26-5	sulfoxide derivative
N-Vanillylnonamide	-	Phytolab	2444-46-4	Amide
Dehydroandrographolide	-	Phytolab	134418-28-3	diterpene
Embelin	-	Phytolab	550-24-3	benzoquinone
Ginkgolic acid C17:1	-	Phytolab	111047-30-4	annacardic acid derivative
Tetrahydropalmatine	-	Phytolab	2934-97-6	benzylisoquinoline alkaloid
6-Shogaol	-	Phytolab	555-66-8	Vanilloid derivative
Jatrorrhizine chloride	-	Phytolab	6681-15-8	benzylisoquinoline alkaloid

Name	Fraction	Source	CAS number	Type of molecule
Trillin	-	Phytolab	14144-06-0	
Withaferin A	-	Phytolab	5119-48-2	steroidal lactone
Chelerythrine	-	Phytolab	34316-15-9	benzylisoquinoline alkaloid
Dehydrocostus lactone	-	Phytolab	477-43-0	sesquiterpene lactone
Epoxybergamottin	-	Phytolab	206978-14-5	coumarin
Gramine	-	Phytolab	87-52-5	indole alkaloid
Tomatidine hydrochloride	-	Phytolab	6192-62-7	triterpene
Physcion	-	Phytolab	521-61-9	anthraquinone
Linderalactone	-	Phytolab	728-61-0	sesquiterpene lactone
Urushiol (15:1)	-	Phytolab	35237-02-6	cardol derivative
Andrograpanin	-	Phytolab	82209-74-3	diterpene
Chelidonic Acid	-	Phytolab	99-32-1	pyran derivative
14-Deoxy 11,14-didehydroandrographolide	-	Phytolab	42895-58-9	diterpene
Eugenitin	-	Phytolab	480-12-6	chromone derivative
Harmine	-	Phytolab	442-51-3	indol akaloid
Trans-Sinapic acid	-	Phytolab	7362-37-0	phenyl propane derivative
Piperine	-	Phytolab	94-62-2	Amide
Linderane	-	Phytolab	13476-25-0	sesquiterpene lactone
Urushiol (15:2)	-	Phytolab	83258-37-1	cardol derivative
Andrographolide	-	Phytolab	5508-58-7	diterpene
Chelidonine	-	Phytolab	476-32-4	benzylisoquinoline alkaloid
12-Deoxywithastramonolide	-	Phytolab	60124-17-6	steroidal lactone
Formosanin C	-	Phytolab	50773-42-7	saponin
Hydrastine	-	Phytolab	118-08-1	benzylisoquinoline alkaloid
Sweroside	-	Phytolab	14215-86-2	secoiridoid
10-Gingerol	-	Phytolab	23513-15-7	Vanilloid derivative
quercetrin-4'-O-Glucoside	-	Phytolab	20229-56-5	flavonoide

Name	Fraction	Source	CAS number	Type of molecule
Solasodine	-	Phytolab	126-17-0	triterpene
Yamogenin	-	Phytolab	512-06-1	triterpene
Diosgenin	-	Phytolab	512-04-9	triterpene
Sinigrin potassium salt	-	Phytolab	3952-98-5	glucosinolate
medicagenic acid	-	Phytolab	599-07-5	aglycone
1,9-Pyrazoloanthrone	-	Sigma	129-56-6	anthrone derivative
pyridoxatin	-	Sigma	135529-30-5	alkaloid
sahandinone	-	Salvia sahendica	-	diterpenoids
12-deoxy-salvipisone	-	Salvia sahendica	-	diterpenoids
7α-Acetoxyroyleanone	-	Salvia sahendica	-	diterpenoids
sahandone	-	Salvia sahendica	-	diterpenoids
Ferruginol	-	Salvia sahendica	-	diterpenoids
cryptotanshinone	-	Perovskia atriplicifolia	-	diterpenoids
PAR 13-5 (Isograndifoliol)	-	Perovskia atriplicifolia	-	diterpenoids
PAR-8 (1hydroxy-cryptotanshinone)	-	Perovskia atriplicifolia	-	diterpenoids
Fathaian-1	-	Hymenocrater elegans	-	diterpenoids
Fathaian-12-7	-	Hymenocrater elegans	-	diterpenoids
Fathaian-3	-	Hymenocrater elegans	-	diterpenoids
Fathaian-F6	-	Hymenocrater elegans	-	diterpenoids
Fathaian-F7	-	Hymenocrater elegans	-	diterpenoids
Fathaian-F10	-	Hymenocrater elegans	-	diterpenoids
Fathaian-F9	-	Hymenocrater elegans	-	diterpenoids
SLE-G1	-	Salvia leriifolia	-	diterpenoids
SLE-E5	-	Salvia leriifolia	-	diterpenoids
SLE-D4	-	Salvia leriifolia	-	diterpenoids
SLE-F3	-	Salvia leriifolia	-	diterpenoids
SLE-D1	-	Salvia leriifolia	-	diterpenoids

Name	Fraction	Source	CAS number	Type of molecule
SLE-B1 (taxodion)	-	Salvia leriifolia	-	diterpenoids
SLE-E9	-	Salvia leriifolia	-	diterpenoids
SLE-F2 (nemerone)	-	Salvia leriifolia	-	diterpenoids
SLH-33-H13 (lupeol)	-	Salvia leriifolia	-	triterpenoids
SLH-16-F1 (3b-Acetoxy-1b-hydroxy-lupeol) new	-	Salvia leriifolia	-	triterpenoids
SLH-13-E15 (3BETA-Acetoxy lupan 20 -ol)	-	Salvia leriifolia	-	diterpenoids
SLH-14-E15 (3b-Acetoxylupan-20-ol)	-	Salvia leriifolia	-	triterpenoids
SLH-15-E16 (3BETA-Acetoxy lupan 20 -ol)	-	Salvia leriifolia	-	triterpenoids
SLH-3-C (Taxodion)	-	Salvia leriifolia	-	diterpenoids
SLH-17-D	-	Salvia leriifolia	-	diterpenoids
Waltherione M	339	*Waltheria Indica*	-	quinolone alkaloids
Waltherione N	321-A	*Waltheria Indica*	-	quinolone alkaloids
(R)-Vanessine	321-B	*Waltheria Indica*	-	quinoline alkaloids
Waltherione P	353	Waltheria Indica	-	quinoline alkaloids
Waltherione O	355	*Waltheria Indica*	-	quinolone alkaloids
Waltherione Q	369-B	*Waltheria Indica*	-	quinolone alkaloids
antidesmone	-	*Waltheria Indica*	-	quinoline alkaloids
waltherione I	-	*Waltheria Indica*	-	quinoline alkaloids
waltherione J	-	*Waltheria Indica*	-	quinoline alkaloids
waltherione L	-	*Waltheria Indica*	-	quinoline alkaloids
waltherione H	-	*Waltheria Indica*	-	quinoline alkaloids
waltherione K	-	*Waltheria Indica*	-	quinoline alkaloids
Flindersine	-		523-64-8	
8-methoxyflindersine	-		35989-00-5	
Ergost-2-en-26-oic acid, 4-(acetyloxy)-5,6:18,20-diepoxy-18,22-dihydroxy-1-oxo-, δ-lactone, (4β,5β,6β,22R,25R)- (9CI)	-	Dunalia brachyacantha	240809-75-0	Withanolides
3-hydroxy-Norvaline	-		2280-42-4	Amino acid derivate

Name	Fraction	Source	CAS number	Type of molecule
Phomalone	-	*Phoma etheridgei*	159768-89-5	phenolic derivative
4a,5,6,7,8,8a-hexahydro-4-hydroxy-4-(hydroxymethyl)-3,4a,8,8-tetramethyl-, (4R,4aS,8aS)-1(4H)-Naphthalenone	-	*Aspergillus ustus*	127681-58-7	Drimane sesquiterpenoids
flindulatin	-	*Waltheria Indica*	New	polyhydroxymethoxyflavonoids
Oxyanin A	-	*Waltheria Indica*	New	polyhydroxymethoxyflavonoids
Chrysosplenol E	-	*Waltheria Indica*	-	polyhydroxymethoxyflavonoids
Vitexicarpin	-	*Waltheria Indica*	-	polyhydroxymethoxyflavonoids
5-Hydroxy-3,4',7-trimethoxyflavone	-	*Waltheria Indica*	New	polyhydroxymethoxyflavonoids

Table S2: List of strains used in this study

Strain	Specices	Parental strain	Plasmid used	Genotype	Reference
SC5314	*C. albicans*	-	-	wild-type	(1)
CAF2-1	*C. albicans*	SC5314		*URA3/ura3*Δ::imm434 *IRO1/iro1*Δ::imm434	(2)
DSY731	*C. albicans*			Azole-susceptible clinical isolate	(3)
DSY732	*C. albicans*	related to DSY731		Azole-resistant clinical isolate	(3)
DSY1841	*C. albicans*			Azole-susceptible clinical isolate	(4)
DSY1843	*C. albicans*	related to DSY1841		Azole-resistant clinical isolate	(4)
SDY1	*C. albicans*	SC5314	pSD1	*erg6*Δ::*SAT1*::FRT/*ERG6*	this study
SDY4	*C. albicans*	SDY1	PSD3	*erg6*Δ::FRT/*erg6*Δ::*SAT1*::FRT	this study
DSY446	*C. albicans*	CAF4.2		*cdr1*Δ::*hisG-URA3-hisG/CDR1*	(5)
DSY448	*C. albicans*	CAF4.2		*cdr1*Δ::*hisG-URA3-hisG/cdr1*Δ::*hisG*	(5)
DSY649	*C. albicans*	CAF4.2		*cdr2*Δ::*hisG-URA3-hisG/CDR2*	(6)
DSY653	*C. albicans*	CAF4.2		*cdr2Δ*::*hisG-URA3-hisG/cdr1Δ*::*hisG*	(6)
DSY463	*C. albicans*	CAF4.2		*ben*Δ::*hisG-URA3-hisG/BEN*	(5)
DSY465	*C. albicans*	CAF4.2		*benΔ*::*hisG-URA3-hisG/benΔ*::*hisG*	(5)
DSY471	*C. krusei*			ATCC 6258	-
DSY472	*C. tropicalis*			ATCC 75	-
DSY473	*C. parapsiolsis*			ATCC 22019	-
DSY562	*C. glabrata*			Azole-susceptible clinical isolate	(7)
DSY565	*C. glabrata*	related to DSY 562		Azole-resistant clinical isolate	(7)
S288C	*S. cerevisiae*			MAT**α** *SUC2 gal2 mal2 mel flo1 flo8-1 hap1 ho bio1 bio6*	-
BY4741	*S. cerevisiae*	S288C		MAT**a** *his3*Δ1 *leu2*Δ0 *met15*Δ0 *ura3*Δ0	-
DSY4743	*S. cerevisiae*	BY4741		MAT***a*** *his3Δ1 leu2Δ0 met15Δ0 ura3Δ0 pdr5*Δ::kanMX4	Euroscarf
DSY5103	*S. cerevisiae*	BY4741		MAT**a** *ura3*Δ0 *leu2*Δ0 *his3*Δ1 *met15*Δ0 *ace2*Δ::kanMX4	Euroscarf
P1	*S. cerevisiae*	DSY4743	pFa6a-His3MX6	MAT***a*** *his3Δ1 leu2Δ0 met15Δ0 ura3Δ0 pdr5*Δ::kanMX4 *msh2*Δ::*HIS3*	this study
IMX581	*S. cerevisiae*	CEN.PK113-7D		MAT**a** *can1*::cas9-natNT2 *ura3-52 TRP1 LEU2 HIS3*	(8)
G132D	*S. cerevisiae*	IMX581		MAT**a** *can1*::cas9-natNT2 *ura3-52 TRP1 LEU2 HIS3 erg6*-G132D	this study

D249G	*S. cerevisiae*	IMX581		MAT**a** *can1*::cas9-natNT2 *ura3-52 TRP1 LEU2 HIS3 erg6*-D249G	this study
RH4237	*S.cerevisiae*	RH448		MAT**α** *erg6*Δ::*LEU2 his4*Δ1 *leu2*Δ*0 lys2*Δ*0 bar1*Δ0 *ura3*Δ*0*	(9)
RH4217	*S.cerevisiae*	RH448		MAT**α** *erg4*Δ::*URA3 his4*Δ1 *leu2*Δ0 *lys2*Δ0 *bar1*Δ0 *ura3*Δ*0*	(9)

Table S3: Primers and plasmids used in this study

Primer name	Sequence (5'->3')	Description
For_msh2_Sc	TTATCTGCTGACCTAACATCAAAATCCTCAGATTAAAAGTCGGATCCCCGGGTTAATTAA	Deletion of MSH2 (pFa6a-His3MX6)
Rev_msh2_Sc	TATCTATCGATTCTCACTTAAGATGTCGTTGTAATATTAAGAATTCGAGCTCGTTTAAAC	Deletion of MSH2 (pFa6a-His3MX6)
p426_CRISPR_rv	GATCATTTATCTTTCACTGCGGAGAAG	Amplification pMel10 linear fragment
p426_CRISPR_fw	GTTTTAGAGCTAGAAATAGCAAGTTAAAATAAGGCTAGTC	Amplification pMel10 linear fragment
ERG6_cri_gRNA_For	TGCGCATGTTTCGGCGTTCGAAACTTCTCCGCAGTGAAAGATAAATGATCTGTTCCATGTCGA CGTGGCTGTTTTAGAGCTAGAAATAGCAAGTTAAAATAAGGCT AGTCCGTTATCAAC	Construction of guideRNA for D249G
ERG6_cri_gRNA__Rev	GTTGATAACGGACTAGCCTTATTTTAACTTGCTATTTCTAGCTCTAAAACAGCCACGTCGACAT GGAACAGATCATTTATCTTTCACTGCGGAGAAGTTTCGAACGCCGAAACATGCGCA	Construction of guideRNA for D249G
ERG6_cri_gRNA_For2	TGCGCATGTTTCGGCGTTCGAAACTTCTCCGCAGTGAAAGATAAATGATCAGAGAGATTGCA AGATTTACGTTTTAGAGCTAGAAATAGCAAGTTAAAATAAGGCTAGTCCGTTATCAAC	Construction of guideRNA for mutation G132D
ERG6_cri_gRNA_Rev2	GTTGATAACGGACTAGCCTTATTTTAACTTGCTATTTCTAGCTCTAAAACGTAAATCTTGCAAT CTCTCTGATCATTTATCTTTCACTGCGGAGAAGTTTCGAACGCCGAAACATGCGCA	Construction of guideRNA for mutation G132D
ERG6_cri_rep_160a_For	ATATGACGAAAACAATCCTGAACATAGAAAGATCGCTTATGAAATTGAACTAGGTGGTGG	Construction of repair fragment for D249G
ERG6_cri_rep_160bs_For	TAGGTGGTGGTATCCCAAAGATGTTCCATGTCGATGTGGCTAGGAAAGCATTGAAGAACT	Construction of repair fragment for D249G
ERG6_cri_rep_160c_Rev	TCATCATCATTGTCCGCCAGGTCTTCGCTAACGAGGACTTCGAAACCACAGTTCTTCAAT	Construction of repair fragment for D249G
ERG6_cri_rep_160a_For2	AGCTTACAAGGCTGGTATTCAAAGAGGCGATTTAGTTCTCGACGTTGGTTGTGGTGTTGG	Construction of repair fragment for G132D
ERG6_cri_rep_160bs_For2	GTGGTGTTGGGGACCCAGCAAGAGAGATTGCTAGATTTACCGGTTGTAACGTCATCGGTC	Construction of repair fragment for G132D
ERG6_cri_rep_160c_Rev2	TTTTTAGCGTAATATTTTGCCTTGGCAATTTGGTAATCGTTATTGTTTAGACCGATGACG	Construction of repair fragment for G132D
Erg6_verif_for	GAGTTTCGCTGCCTCGATAG	Sequencing primer for G132D
Erg6_verif_rev	TCATTGTCCGCCAGGTCTTC	Sequencing primer for D249G
ERG6_5For_KPN1	CGCGGTACCACAACGATCTATTCTTCTCG	Deletion of *ERG6* first allele (pSD1 and pSD4)
ERG6_5Rev_Xho1	CGCCTCGAGGTTAATGAGTTGAATCTTAG	Deletion of *ERG6* first allele (pSD1 and pSD4)
ERG6_3For_SacII	CGCCCGCGGCGTGTATCTATTCATAGAGG	Deletion of *ERG6* first allele (pSD1)
ERG6_3Rev_SacI	CGCGAGCTCGACATTGATAAGGCTAAGAG	Deletion of *ERG6* first allele (pSD1)
ERG6_3CDSFor_SacII	CGCCCGCGGAGATTTGGCTGATGTTGATG	Deletion of *ERG6 second* allele (pSD4)
ERG6_3CDSRev_SacI	CGCGAGCTCCCCAACATAAACTCACCCTG	Deletion of *ERG6* second allele (pSD4)
ACT1_for	GCATCACACTTTTTACAAT	qPCR
ACT1_rev	AAACATAATTTGAGTCATCTTT	qPCR

Primer name	Sequence (5'->3')	Description
ACT1_probe	FAM-TTGCTCCAGAAGAACATCCAGT-TAMRA	qPCR
ERG4_for	TCAAATGTGCCAATGGTTCT	qPCR
ERG4_rev	AGCCCAAGTCAATGTTTGAA	qPCR
ERG4_probe	FAM- TCTGGCATAAACGTACCACCCATCA-TAMRA	qPCR
ERG6_for	ATGAACATTTCTTGGCCCAT	qPCR
ERG6_rev	CCAACAATTTCACAATCAGTAAATC	qPCR
ERG6_probe	FAM-CCAGGACCACCTACACCACAACCA-TAMRA	qPCR
ERG11_for	ATTGTTGAAACTGTCATTG	qPCR
ERG11_rev	CCCCTAATAATATACTGATCTG	qPCR
ERG11_probe	FAM- TTTGTCCCTTAGTGTTACACA-TAMRA	qPCR
CDR1_for	ATGACTCGAGATATTTTGATA	qPCR
CDR1_rev	TTAACAGCAATGGTCTTTA	qPCR
CDR1_probe	FAM-CATTATGAGACCTGGTGAACTTACT-TAMRA	qPCR
CDR2_for	TAGATATTTGAGCCACATG	qPCR
CDR2_rev	TTGGCATTGAAATTTTCG	qPCR
CDR2_probe	FAM- TTAGTCCATTCAACGGCAACATTAG-TAMRA	qPCR

Plasmid	Characteristics	Reference
pSD1	5'-UTR and 3'-UTR of *CaERG6* into pSFS2a (KpnI,XhoI; SacI,SacII)	(10)
pSD4	5-'UTR and 3'-CDS end of *CaERG6* into pSFS2a (KpnI,XhoI; SacI,SacII)	(10)
pFa6a-His3MX6	Plasmid carrying the *HIS3* selection marker, used to delete *MSH2*	(11)
pMel10	Plasmid carrying the *KIURA3*-selection marker, expression of gRNA	(8)

Table S4: Activity of tomatidine against a collection of *Candida* spp.

Species	Strain [a)]	MIC [b)]		MIC50		MIC90	
		Tomatidine (µM)	Fluconazole (µg/ml)	Tomatidine (µM)	Fluconazole (µg/ml)	Tomatidine (µM)	Fluconazole (µg/ml)
C. albicans	DSY227	0.625	0.5	0.3125	0.5	2.5	2
	DSY281	0.3125	0.5				
	DSY285	0.3125	0.5				
	DSY288	0.3125	1				
	DSY290	2.5	0.5				
	DSY294	0.3125	0.5				
	DSY299	0.3125	0.25				
	DSY297	0.3125	2				
	SC5314	0.625	0.5				
C. krusei	DSY2293	0.625	32	1.25	32	>40	32
	DSY2294	>40	32				
	DSY2300	0.625	32				
	DSY2302	1.25	32				
	DSY2616	>40	32				
	DSY2617	1.25	32				
	DSY2618	1.25	32				
	DSY471	0.625	32				
C. tropicalis	DSY381	0.3125	0.5	1.25	8	5	128
	DSY2274	2.5	2				
	DSY2310	1.25	2				
	DSY2336	1.25	16				
	DSY2374	0.3125	8				
	DSY2897	1.25	4				
	DSY2825	5	128				
	DSY826	0.15	2				
	DSY472	5	128				

[a)] Strains are from the laboratory collection (“DSY” prefix). Strain identities were confirmed by MALDI-TOF approaches.

[b)] Tomatidine MICs were determined as described in Material and Methods with YNB buffered at pH 7.0. Fluconazole MICs were determined according to EUCAST protocols.

Supplementary Figures

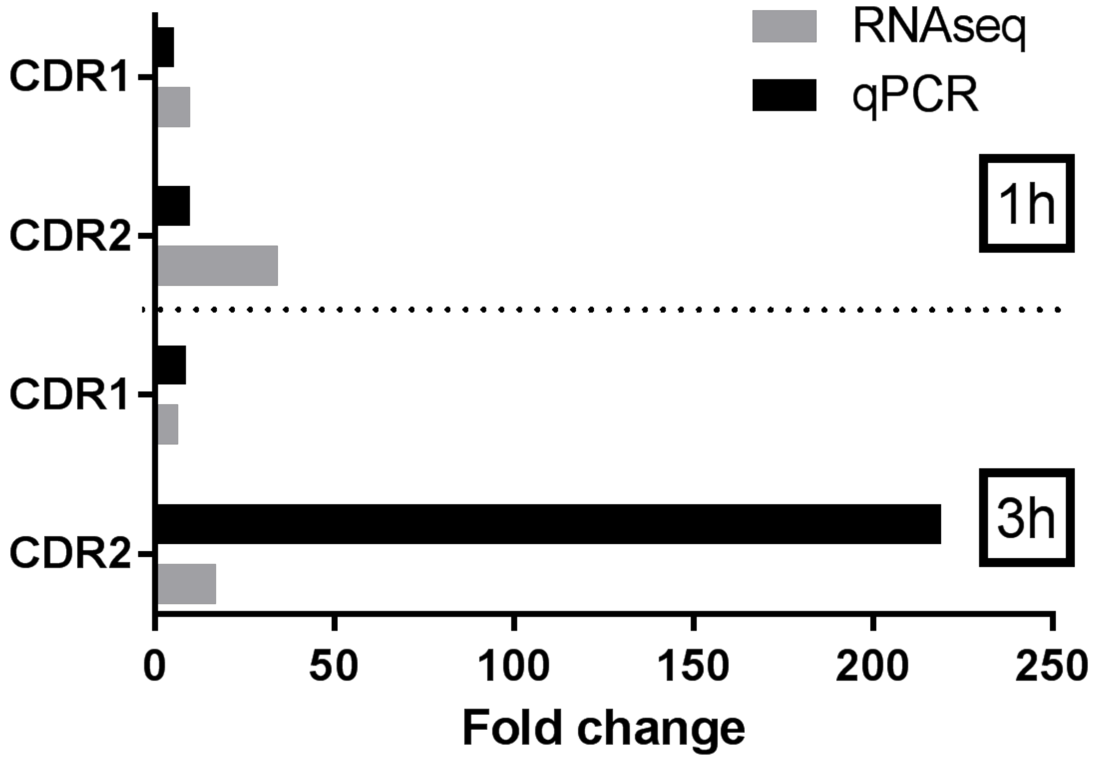

Figure S1: qPCR analysis of *CDR1* and *CDR2* expression in *C. albicans* cells after 1- and 3h tomatidine treatment. Fold-change increase in expression compared to untreated cells is represented in a barplot. T-test analyses were performed between treated and untreated group for each gene relative expression and significant differences detected (all p-value < 0.05).

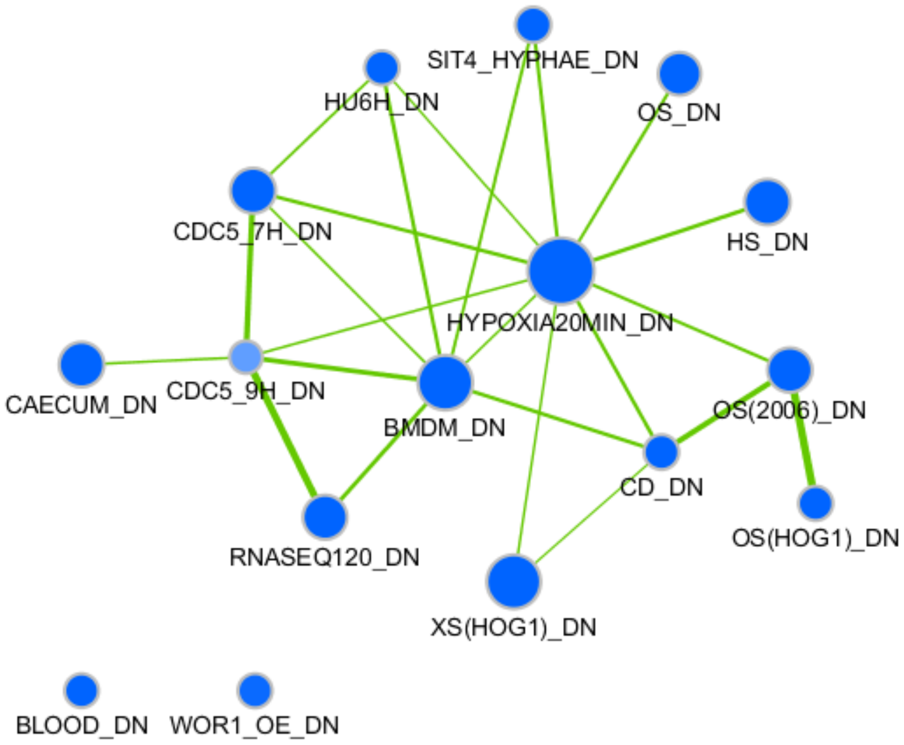

Figure S2: Gene set enrichment analysis (GSEA) of *C. albicans* genes regulated by tomatidine with stress-related transcriptional data.

The list of stress-regulated genes was adapted from published transcriptional data (12) (see supplementary file S2, CandidaL_exp_dataL2mod.gmt). Tomatidine regulated genes (3h) were ranked according to their fold-change. The list was then imported into the GSEA software. Analysis parameters were as follows: norm, meandiv; scoring_scheme, weighted; set_min, 15; nperm, 1000; set_max, 500. GSEA results were uploaded into Cytoscape 3.0 with the following parameters: p-value cut-off, 0.01; FDR q-value, 0.05. Red nodes represent enriched gene lists in upregulated (UP) genes from the GSEA. Blue nodes represent enriched gene lists in downregulated (DN) genes from the GSEA. Nodes are connected by edges when overlaps exist between nodes. The size of nodes reflects the total number of genes that are connected by edges to neighbouring nodes. The labels of the list (*C. albicans* stress- and growth conditions) are indicated next to the nodes (details in Supplementary File S2).

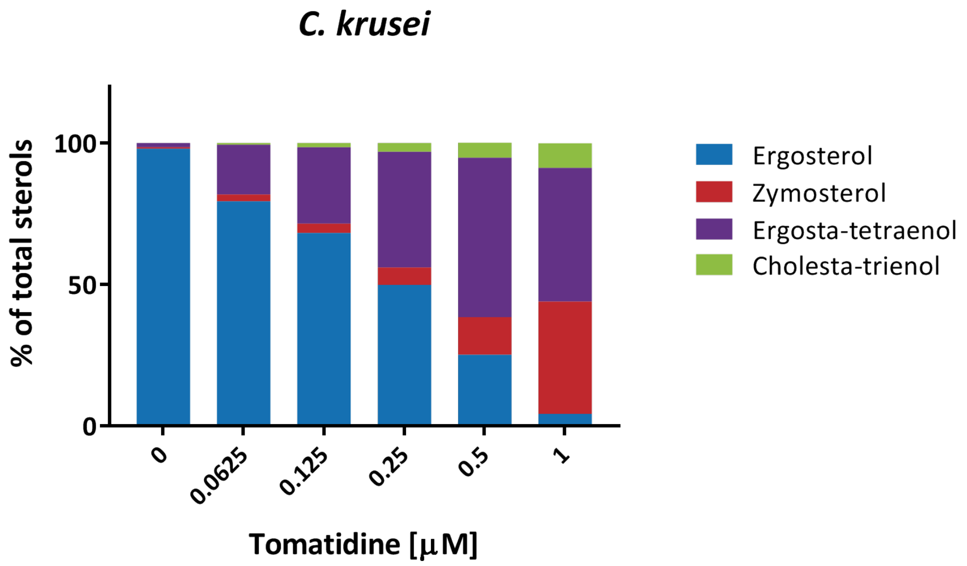

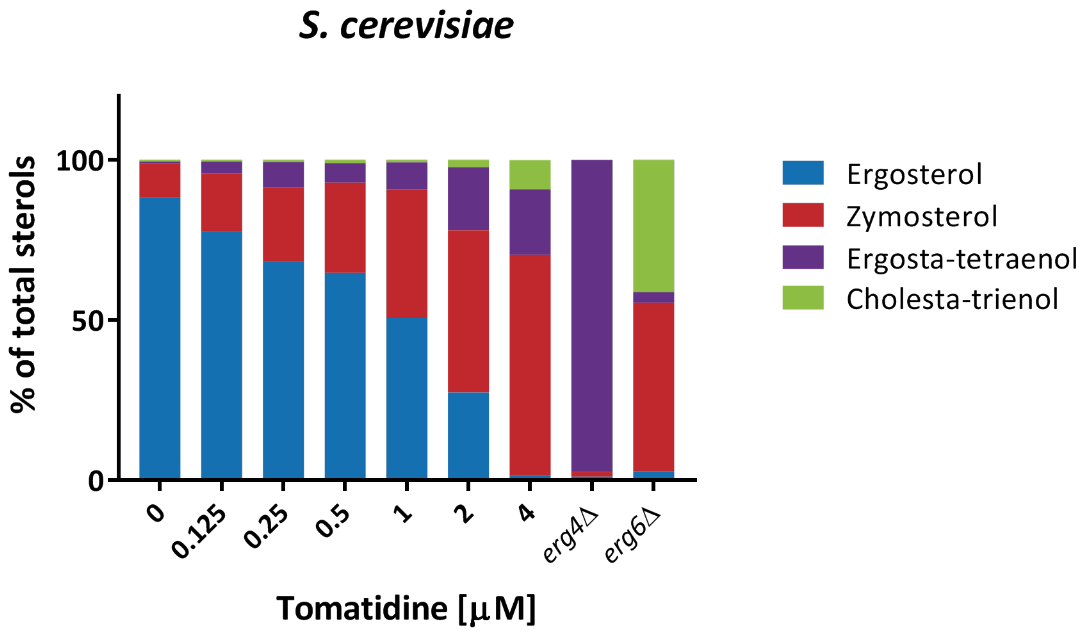

Figure S3: GC-MS analysis of sterol contents of *C. krusei* and *S. cerevisiae* cells after tomatidine exposure. GC-MS analysis of cells exposed to tomatidine with the percentage of the different sterols are shown in a bar plot. Bar colors correspond to sterol molecules illustrated in the left panel of Figure 3A where the last steps in the ergosterol biosynthetic pathway and an alternative pathway after Erg6 inhibition are shown. The sterol profiles of *S. cerevisiae erg4*Δ (RH4217) and *erg6*Δ (RH4237) mutants are also shown. Experiments were repeated (duplicates) and gave similar results.

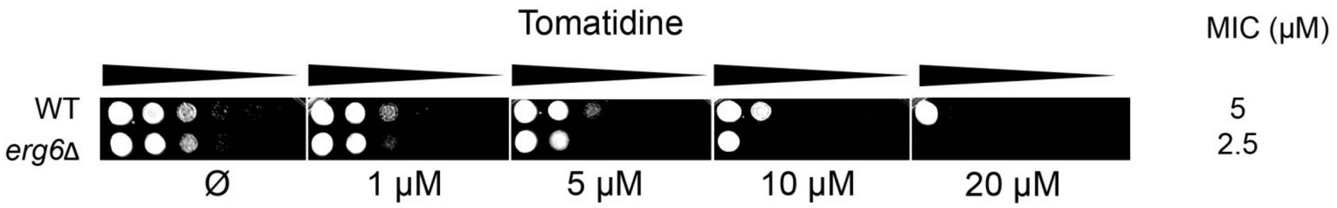

Figure S4: Susceptibility assay of the *S. cerevisiae ERG6* deletion strain.

WT (BY4741) and *erg6Δ* strain (RH4237) were subjected to serial dilution susceptibility assay on YPED plate containing indicated concentration of tomatidine and to standard MIC determination assay in YNB-CSM pH 7 media.

A

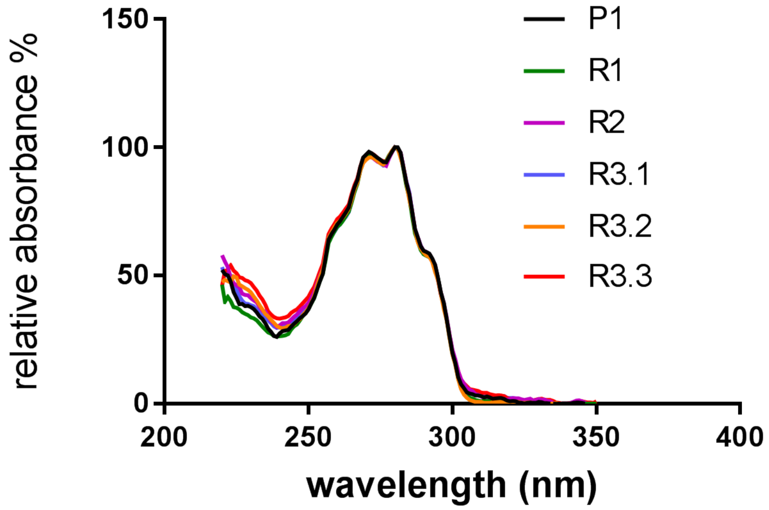

B

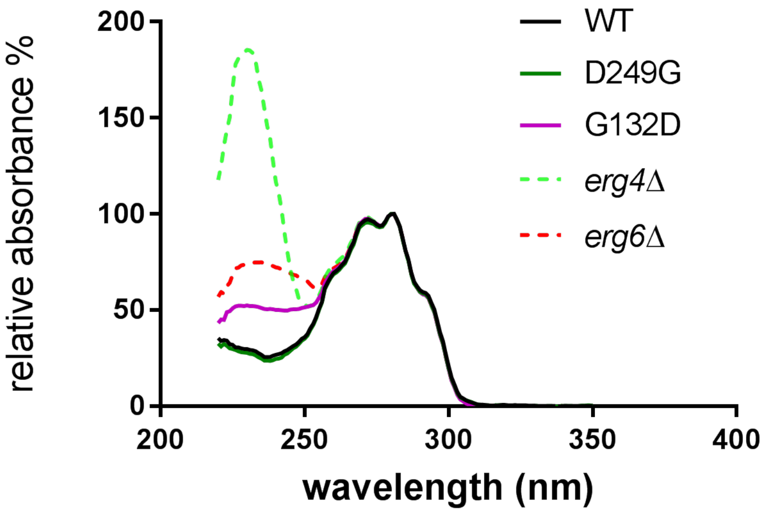

Figure S5: UV spectrophotometric sterol profiles of tomatidine-resistant strains (A) and corresponding *ERG6* point-mutated strains (B). *S. cerevisiae* cells were grown overnight in YPED media, sterol extracted and spectral profiles between 220 and 350 were determined (as described in Materials and Methods). To normalize the spectra, a relative absorbance was calculated with 100% absorbance corresponding to the 281 nm peak. Strains represented are: **(A)** Tomatidine-resistant strains and the parental susceptible strain; **(B)** WT (IMX581), the two *ERG6* point-mutated strains (D249G and G123D) and as control *erg6*Δ (RH4237) and *erg4*Δ (RH4217).

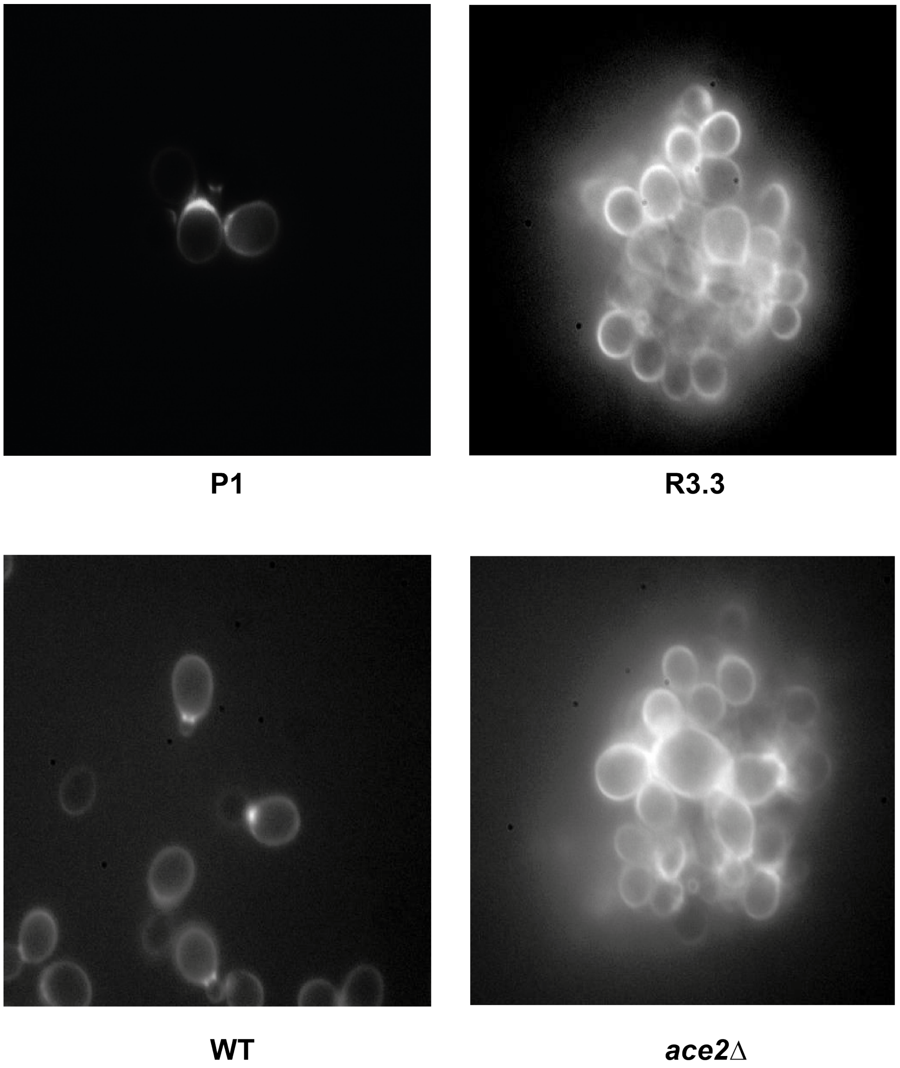

Figure S6: Microscopic analysis of the effect of *ACE2* deletion on cell morphology.

Samples were taken in exponential phase (OD_{540} 0.4) from culture grown in YPED media and resuspended in PBS as described in Materials and Methods. Upper panel: Calcofluor White staining of parental strain P1 and tomatidine-resistant strain R3.3. P1 has a similar morphology to the wild type strain. Microscopic observation of R3.3 revealed a multicellular clumping phenotype typical for *ace2*Δ mutant as shown in the right lower panel. Images are representative for the entire culture.

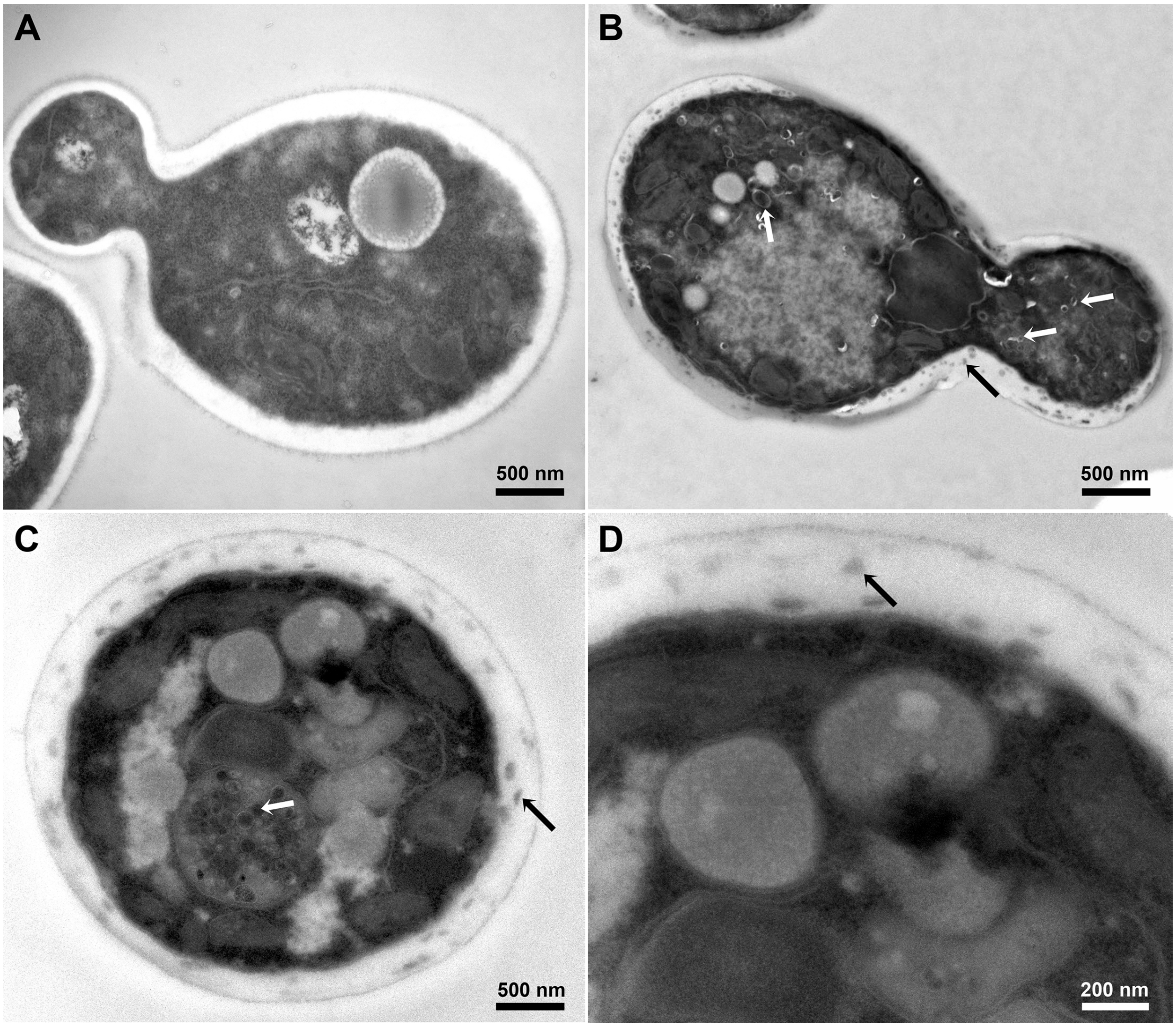

Figure S7: Ultrastructure of *C. albicans* wild-type cells with and without treatment with tomatidine observed by transmission electron microscopy (TEM)

(A) Control with DMSO treatment showing intact membrane, organelles, wall space and numerous ribosomal particles. **(B)** Cell treated with miconazole for 18 h. Black arrow shows dark material in the wall space and white arrows dark lipid-like corpuscules. **(C)** Cell treated with tomatidine (10 µM) for 18 h. Black arrow shows dark material in the wall space and white arrow dark lipid-like corpuscules in a vacuole. **(D)** Details of C panel showing the dark material accumulating in the cell wall space.

References

1. Gillum AM, Tsay EY, Kirsch DR. Isolation of the Candida albicans gene for orotidine-5'-phosphate decarboxylase by complementation of S. cerevisiae ura3 and E. coli pyrF mutations. Mol Gen Genet. 1984;198(2):179-82.
2. Fonzi WA, Irwin MY. Isogenic strain construction and gene mapping in Candida albicans. Genetics. 1993;134(3):717-28.
3. Coste A, Selmecki A, Forche A, Diogo D, Bougnoux ME, d'Enfert C, et al. Genotypic evolution of azole resistance mechanisms in sequential Candida albicans isolates. Eukaryot Cell. 2007;6(10):1889-904.
4. White TC, Pfaller MA, Rinaldi MG, Smith J, Redding SW. Stable azole drug resistance associated with a substrain of Candida albicans from an HIV-infected patient. Oral Dis. 1997;3 Suppl 1:S102-9.
5. Sanglard D, Ischer F, Monod M, Bille J. Susceptibilities of *Candida albicans* multidrug transporter mutants to various antifungal agents and other metabolic inhibitors. Antimicrob Agents Chemother. 1996;40(10):2300-5.
6. Sanglard D, Ischer F, Monod M, Bille J. Cloning of Candida albicans genes conferring resistance to azole antifungal agents: characterization of CDR2, a new multidrug ABC transporter gene. Microbiology. 1997;143 (Pt 2):405-16.
7. Sanglard D, Ischer F, Calabrese D, Majcherczyk PA, Bille J. The ATP binding cassette transporter gene CgCDR1 from Candida glabrata is involved in the resistance of clinical isolates to azole antifungal agents. Antimicrob Agents Chemother. 1999;43(11):2753-65.
8. Mans R, van Rossum HM, Wijsman M, Backx A, Kuijpers NG, van den Broek M, et al. CRISPR/Cas9: a molecular Swiss army knife for simultaneous introduction of multiple genetic modifications in Saccharomyces cerevisiae. FEMS Yeast Res. 2015;15(2).
9. Friant S, Meier KD, Riezman H. Increased ubiquitin-dependent degradation can replace the essential requirement for heat shock protein induction. EMBO J. 2003;22(15):3783-91.
10. Reuss O, Vik A, Kolter R, Morschhauser J. The SAT1 flipper, an optimized tool for gene disruption in Candida albicans. Gene. 2004;341:119-27.
11. Longtine MS, McKenzie A, 3rd, Demarini DJ, Shah NG, Wach A, Brachat A, et al. Additional modules for versatile and economical PCR-based gene deletion and modification in Saccharomyces cerevisiae. Yeast. 1998;14(10):953-61.
12. Amorim-Vaz S, Tran Vdu T, Pradervand S, Pagni M, Coste AT, Sanglard D. RNA Enrichment Method for Quantitative Transcriptional Analysis of Pathogens In Vivo Applied to the Fungus Candida albicans. MBio. 2015;6(5):e00942-15.
